# Perceptions and attitudes towards obesity and metabolic and bariatric surgery for weight loss in children and adolescents: a survey among a subset of Chinese residents

**DOI:** 10.1186/s12889-026-28495-7

**Published:** 2026-07-18

**Authors:** YuRou Yang, Wenhuan He, GuoQiang Wang, Biao Huang

**Affiliations:** 1 Guangzhou Medical University Affiliated Dental Hospital, Guangzhou, China; 2 the Second Affiliated Hospital of Guangzhou Medical University, Guangzhou, China

**Keywords:** Metabolic and bariatric surgery, Childhood and adolescent obesity, Perceptions and attitudes, Cross-sectional survey, Health literacy

## Abstract

**Background:**

The prevalence of obesity among children and adolescents and the number of cases of bariatric surgery among children and adolescents is increasing; the discussion on weight loss among children and adolescents is becoming polarized.

**Objectives:**

To investigate the prevalence of overweight and obesity among a sample of Chinese children and adolescents and the perceptions and attitudes of a subset of Chinese residents towards obesity, weight loss, and MBS for weight loss in children and adolescents.

**Methods:**

Cross-sectional survey among a subset of Chinese residents, collected from August 5, 2022 to October 30, 2022. Participants were a subset of Chinese population, including adults, children, and adolescents.

**Results:**

A total of 3015 questionnaires were returned. Children and adolescents with overweight and obesity accounted for 16.6% and 9.4%. Gender, education, occupation and age were associated with the perceptions and attitudes of Chinese residents toward obesity and weight loss in children and adolescents; age, occupation and BMI were associated with the perceptions and attitudes toward MBS in children and adolescents. There are 10.8% of the respondents thought MBS was the most suitable method for children and adolescents, and 34.7% were unwilling to have MBS for their children. The percentage of respondents who thought it was appropriate for children and adolescents to undergo bariatric surgery at the age of 12–18 was 18.6%; 60.96% said they did not know enough about the surgery; most were aware that the effects of the surgery included weight loss (84.78%), improved self-confidence/ improved self-image (78.97%), improved disease (e.g., reduced drug use) (70.3%) and better performance in interpersonal interactions (51.70%).

**Conclusions:**

A subset of Chinese residents are not fully aware of the harm of obesity among children and adolescents and its related complications. They have insufficient knowledge and trust in MBS and underestimate the benefits of the procedure. More health education is still required in these areas.

**Supplementary Information:**

The online version contains supplementary material available at https://doi.org/10.1186/s12889-026-28495-7.

## Key points

1. A small portion children and adolescents with obesity and overweight in our survey.

2. A subset of Chinese residents do not know enough about the causes and hazards of obesity in children and adolescents.

3. A subset ofChinese residents need more knowledge and trust in metabolic and bariatric surgery and its benefits for obesity and related metabolic disorders.

**Supplementary Information:**

The online version contains supplementary material available at https://doi.org/10.1186/s12889-026-28495-7.

## Introduction

Along with economic growth and urbanization, childhood and adolescent obesity has become an increasingly common public health problem worldwide. Some scholars estimate that the global overweight rate of children and adolescents aged 5–17 will rise from 13.9% in 2010 to 15.8% in 2025. The number of overweight children and adolescents in China will reach 48.5 million in 2025 [1]. In 2015, the overweight and obesity rates among children and adolescents in China increased to 16.23% and 10.75% [2], respectively. There are 15.3% of children in the top five cities in China were in obesity in 2017 (19.2% of boys and 11.2% of girls) [3]. Related studies show that 65%-70% of children and adolescents are still in obesity by adulthood [4–6].

Obesity-associated medical problems in children and adolescents include arterial hypertension, dysglycemia and non-alcoholic steatohepatitis (NASH), polycystic ovary syndrome (PCOS) in girls, and mental disorders such as depression or attention deficit disorder [7]; and increase the risk of cancer in adulthood [8, 9] and the risk of cardiovascular morbidity and mortality in midlife [10].

Metabolic and bariatric surgery (MBS) has been widely proven safe and effective in adults. Surgery for weight loss in children and adolescents with obesity also attracts increasing attention [11–13]. Evidence suggests that surgery in children and adolescents is safe and effective [14] and is more likely to alleviate obesity-related diseases than surgery in adulthood [15]. However, MBS in children and adolescents is rarely reported in China, which may be related to the lack of popularity of the procedure, parents’ lack of understanding and low trust in bariatric surgery, the traditional Chinese concept of avoiding medical treatment and the low regard for obesity in children and adolescents. To clarify the specific reasons, we conducted a national survey on Chinese residents, aiming to understand the current prevalence of childhood and adolescent obesity in China, the perceptions and attitudes of Chinese residents towards childhood and adolescent obesity, and the perceptions and attitudes towards MBS in children and adolescents, to contribute to better public health education, prevention and control of childhood and adolescent obesity and to make the best medical decisions at the macro level.

## Participants and methods

### Participants of the study

The participants of this study were a subset of Chinese residents of all ages who cooperated voluntarily, were able to use electronic devices correctly, and understood the content of the questionnaire.

### Research methodology

The researchers drew on relevant professional literature [16, 17] to develop a preliminary questionnaire. In terms of content validity, five experts (including one clinical expert in metabolic and bariatric surgery, two metabolic and bariatric case managers, and two public health/nursing researchers) were invited to review and collectively evaluate the questionnaire items in terms of relevance, comprehensiveness, and clarity of expression. Revisions were made item by item based on the experts’ feedback, and finally the experts rated the items. The S-CVI/Ave was 0.88, indicating good content validity. After obtaining ethical approval (KY-2022-252), a pilot survey was conducted among 35 Chinese residents to assess their understanding of the wording and phrasing of the questions. Following focus group discussions, revisions were made based on selected suggestions, resulting in the final version of the questionnaire (Appendix 1). Regarding reliability, since this questionnaire is a descriptive survey and most items involve single factual variables (such as demographic characteristics and knowledge accuracy), rather than measuring latent psychological traits as in a standardized scale, reliability tests based on internal consistency (Cronbach’s α) are not applicable. Convenience sampling was used in this study, and the questionnaire was distributed through an online questionnaire system for Chinese residents to complete online from August 2022 to October 2025, using an online questionnaire system is called Wen Juan Xing (https://www.wjx.cn), the respondents voluntarily scan the QR code to enter the questionnaire interface.According to the sample size calculation formula for simple random sampling n = Z2α/2×p×(1 − p)/d2, with a 95% confidence level, a margin of error of 3%, and considering a 20% non-response rate, the minimum sample size for this study is finally determined to be 1,281.

The questionnaire consisted of four parts, include 33 items. The first part was general demographic information of participants, including gender, age, education, occupation, height, weight, and self-evaluation of weight. The second part was general demographic information of children completed by parents, and additional information on children’s lifestyle and birth weight was collected. The third part was to assess perceptions and attitudes toward obesity and weight loss in children and adolescents. The fourth part was to assess the attitudes and perceptions towards MBS performed on children and adolescents. Those without children were not required to answer all the questions, and respondents can return to change the answer before submitting it. No financial or other material incentives were offered to the respondents. This study followed the Checklist for Reporting Results of Internet E-Surveys (CHERRIES) [18](Appendix 2). The study design is shown in eFigure 1.

#### Data cleaning

During the survey implementation phase, all interviewers received standardized training and were provided with standardized instructions; mandatory fields and logic jumps were configured in the electronic questionnaires to prevent omissions and errors; for paper questionnaires, a two-person data entry and verification process was implemented to ensure data accuracy. During the data collection phase, the electronic questionnaire backend monitored response progress in real time, flagging and reviewing questionnaires with completion times that were either too short (< 180 s) or too long (> 3,600 s). Invalid questionnaires were excluded if they contained repetitive answers or had missing values for key variables exceeding 20%.

### Statistical analysis

After the survey, data were exported from the online questionnaire system and double-entered by two researchers using a computer respectively to ensure the accuracy of the data. The unqualified questionnaires were also removed. All data were processed using SPSS 24.0 statistical software, and the measurement data were expressed as mean ± standard deviation; the count data were expressed as rates and percentages. The statistics of categorical data were tested by the chi-square test or Fisher’s exact probability method, and the difference was considered statistically significant at *P* < 0.05.

## Results

### General information of the respondents

The total number of respondents was 3100, with 85 questionnaires found to be substandard. 3015 were complete and effective (1304 males, 43.3%; 1711 females, 56.7%); age ranged from 12 to 75 years old (mean ± standard deviation: 31.37 ± 8.73); more than 87.7% of the respondents had a bachelor’s degree or higher; 42% of the respondents were parents (23.1% had one child, 17.1% had two or more children); occupations of the respondents include education (13.7%), service industry (11.4%), healthcare (11.1%), etc.; 52.6% of respondents’ per capita monthly household income is > 9000 Chinese Yuan (CNY); body mass index (BMI) of the respondents was 22.45 ± 3.969 kg/m2, with men (23.66 ± 3.613 kg/m2) slightly higher than that of women (21.53 ± 3.982 kg/m2); 60% of the respondents’ BMI was within the normal range (18.5 ≤ BMI < 24.0), 28.1% were overweight (20.8%) or in obesity (7.3%). Details are shown in Table 1.

**Table 1:** General demographic data

	*N*(%)	Means ± SD		BMI Means ± SD
1.sex				
Male	1304(34.2)		23.66 ± 3.613	
Female	1711(56.7)		21.53 ± 3.982	
2.Age(years)		31.37 ± 8.729		
< 18	54(1.7)			
18–29	1410(46.7)			
30–39	1037(34.3)			
40–49	384(12.7)			
≥ 50	130(4.3)			
3.Education				
Junior high school and below	92(3.0)			
High school or junior college	277(9.1)			
Undergraduate or college	1988(65.9)			
Master and above	658(21.8)			
4.Marital status				
Unmarried	1384(45.9)			
Married	1587(52.6)			
Divorced	40(1.3)			
Widowed	4(0.1)			
5.Occupation				
Finance	310(10.2)			
Education	414(13.7)			
Healthcare	332(11.1)			
Media, Arts	134(4.4)			
Industrial technology related	238(7.8)			
Science, information technology-related	266(8.9)			
Agriculture, forestry, fishery and water production	70(2.3)			
Service industry	344(11.4)			
Transportation Equipment	38(1.2)			
Self-employed	154(5.1)			
Military related	7(0.2)			
Student	284(9.4)			
Unemployed	78(2.5)			
Retired	32(1.0)			
Full-time Mom/Dad	98(3.2)			
Other	216(6.8)			
6.Monthly per capita family income(yuan)				
< 3000	163(5.4)			
3000–6000	595(19.7)			
6001–9000	671(22.2)			
> 9000	1586(52.6)			
7.BMI(kg/m^2^)				
< 18.5	335(11.1)	22.45 ± 3.969		
18.5–23.9	1829(60.7)			
24-27.9	630(20.9)			
≥ 28	221(7.3)			
8.Evaluation of self				
Thin	468(15.5)			
Standard	1443(47.8)			
Overweight	819(27.1)			
Obesity	285(9.4)			
9.Whether you have received MBS				
Yes	34(1.1)			
No	2980(98.8)			
10.Have children				
No	1803(59.8)			
Have one	697(23.1)			
Have two or more	514(17.1)			

### General information of children and adolescents aged 2–18 years

There were 951 out of 3015 respondents provided general information about children and adolescents aged 2–18.According to relevant standards, the body mass index (BMI) of 951 children and adolescents was categorized [19]0.16.6% and 9.4% were overweight and obesity. Children and adolescents aged 12–18 years (61.8%) and parents with a bachelor’s degree or higher (70.0%) had the highest standard BMI rates; children’s BMI was positively correlated with their birth weight (*R* = 0.1006, *P* = 0.0019); it was also positively correlated with their parents’ BMI (*R* = 0.2145, *P* < 0.001). Details are shown in Table 2.

**Table 2:** BMI categories by demographic variables

Variables	BMI of children(*N* = 951) *N*(%)				Means ± SD	*P* value	Coefficient of association
	Thin	Standard	Overweight	Obesity			
	188(19.8)	515(54.2)	159(16.6)	89(9.4)	23.01 ± 3.611		
Sex of children						0.188	0.071
Male	113(21.9)	278(53.9)	77(14.9)	48(9.3)			
Female	75(17.2)	237(54.5)	82(18.9)	41(9.4)			
Age(years)						0.046	0.041
2–6	78(22)	192(54.1)	48(13.5)	37(10.4)	8.537 ± 4.376		
7–12	82(20.7)	200(50.4)	78(19.6)	37(9.3)			
12–18	28(14.1)	123(61.8)	33(16.6)	15(7.5)			
Education						0.017	0.018
Junior high school and below	156(21.7)	369(51.4)	119(16.6)	74(10.3)			
High school or junior college	14(10.1)	93(67.4)	25(18.1)	6(4.3)			
Undergraduate or college	15(20.0)	39(52.0)	13(17.3)	8(10.7)			
Master and above	3(15.0)	14(70.0)	2(10.0)	1(5.0)			
Lifestyle						0.838	
Living with parents	164(19.8)	448(54.0)	142(17.1)	76(9.2)			
Boarding at school	17(20.2)	48(57.1)	11(13.1)	8(9.5)			
Living with grandparents or relatives	6(20.0)	15(50.0)	5(16.7)	4(13.3)			
Living alone or with apartner or friend	0(0.0)	4(66.7)	1(16.7)	1(16.7)			
Living with host family	1(100)	0(0.0)	0(0.0)	0(0.0)			
Birthweight						> 0.05	0.100
Normal	186(98.4)	495(96.3)	152(95.6)	87(97.8)	3.286 ± 0.423		
Low weight	1(0.5)	0(0.0)	0(0.0)	0(0.0)			
Huge baby	2(1.1)	19(3.7)	7(4.4)	2(2.2)			
BMI of parents						> 0.001	0.213
Thin	23(39.7)	29(50)	4(6.9)	2(3.4)			
Standard	122(21.6)	320(56.5)	86(15.2)	38(6.7)			
Overweight	33(13.1)	134(53.4)	53(21.1)	31(12.4)			
Obese	10(13.2)	32(42.1)	16(21.1)	18(23.7)			

The analysis revealed that only 52% of parents could correctly evaluate their children’s body size, and 38.2% underestimated their children’s body size. Notably, it is worth noting that there was a difference between fathers’ and mothers’ assessment of their children’s body size (χ^2^ = 181.41 *P* < 0.001), with mothers (56.6%) being more accurate in assessing their children’s body size than fathers (47.8%). The older parents are more likely to overestimate the size of their children (χ^2^ = 20.75, *P* = 0.001). Details are shown in *eTable 1*.

### Perceptions and attitudes of respondents towards obesity and weight loss in childhood and adolescents

This section includes seven questions (Q1-Q7). There were differences in the level of awareness of BMI by gender, education, age, occupation, and household income (Table 3). Females and people with high education had higher awareness. The best ability to calculate BMI was among those aged 18–29 (50.4%), working in health care (80.7%), and with household income > 9000 Chinese Yuan (51.1%) (*eTable 2 and eTable 3*). The more females (85.9%), highly educated (85.3%), engaged in health care (80.7%), and with a standard BMI (83.1%), the more inclined they were to consider obesity as a disease for adolescents (*eTable 4*).

**Table 3:** Correlation between different questions and factors and differences between factor groups

Variables	Gender	Education	Age(years)	Careers	Monthly per capita family income(yuan)	BMI (kg/m^2^)
Q1	*P* = 0.000 0.089	*P* = 0.000 0.306	*P* = 0.000 0.089	*P* = 0.000 0.275	*P* = 0.001 0.129	*P* = 0.068 0.049
Q2	*P* = 0.000 0.071	*P* = 0.000 0.251	*P* = 0.007 0.069	*P* = 0.000 0.294	*P* = 0.001 0.098	*P* = 0.753 0.02
Q3	*P* = 0.000 0.127	*P* = 0.000 0.155	*P* = 0.006 0.084	*P* = 0.000 0.156	*P* = 0.119 0.058	*P* = 0.020 0.07
Q4	*P* = 0.000 0.132	*P* = 0.000 0.170*	*P* = 0.000 -0.144*	*P* = 0.051 0.020	*P* = 0.245 0.017*	*P* = 0.001 -0.125*
Q5	*P* = 0.000 0.140	*P* = 0.000 0.079*	*P* = 0.006 -0.04*	*P* = 0.000 0.256	*P* = 0.112 0.03*	*P* = 0.377 0.01*
Q6	*P* = 0.000 0.115	*P* = 0.000 0.117	*P* = 0.000 0.123	*P* = 0.000 0.305	*P* = 0.004 0.08	*P* = 0.208 0.053
Q8	*P* = 0.000 0.175	*P* = 0.004 0.027*	*P* = 0.075 -0.36*	*P* = 0.000 0.306*	*P* = 0.206 0.009*	*P* = 0.083 0.056*
Q9	*P* = 0.000 0.09	*P* = 0.517 0.01	*P* = 0.000 0.132	*P* = 0.000 0.140	*P* = 0.037 0.053	*P* = 0.001 0.485
Q11	*P* = 0.083 0.032	*P* = 0.000 0.033	*P* = 0.360 0.129	*P* = 0.000 0.122	*P* = 0.230 0.038	*P* = 0.001 0.466

The correlation coefficient defaults to coefficient of association

Q1: Do you know the body mass index(BMI)?

Q2: Do you know how to calculate BMI?

Q3: Do you think obesity is a disease for children and adolescents?

Q4: What factors do you think may lead to obesity in children and adolescents?

Q5: Which health problems do you think obesity may lead to in children and adolescents?

Q6: Do you think obesity is more harmful to children and adolescents than adults?

Q8: What do you think about the role of metabolic and bariatric surgery for children and adolescents include

Q9: If your child is overweight or obese and traditional diet-exercise treatment is not effective, will you let your child undergo metabolic and bariatric surgery?

Q11: If your child has obesity-related problems between the ages of 7 and 18, and they have a strong desire to undergo metabolic and bariatric surgery, would you agree to undergo surgery?

Those marked with"*” are coefficient of rank correlation

**Fig. 1:**
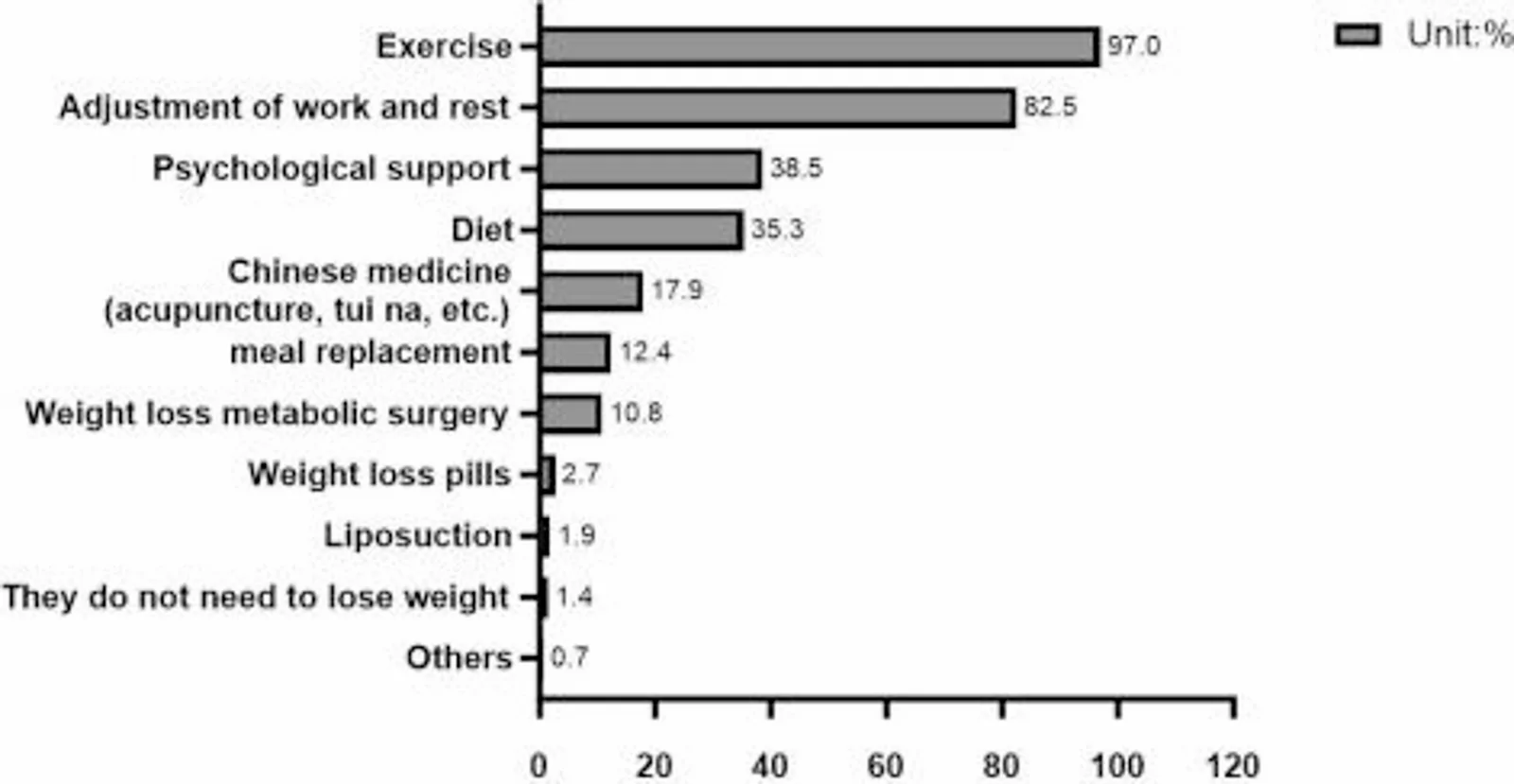
The most suitable way considered by the public for children and teenagers to lose weight

Regarding “factors causing obesity in children and adolescents” and “complications caused by obesity”, and the analysis found that: the higher the awareness scores of females and people with higher education; the higher the awareness level of respondents older than 18 years old.The difference between different education and age groups is statistically significant, but the correlation is not high (*eTables 5 and eTables 6*). Those who are females (33.8%), engaged in health care, above high school with higher education, below 50 years old with older age, and 3000 per capita income with a higher income tend to think that obesity in children and adolescents is more harmful than obesity in adults (*eTable 7*). 97% believed that the most suitable weight loss intervention for children and adolescents was physical exercise, and only 10.8% chose MBS for weight loss; 1.4% of the respondents thought that there was no need for weight loss in children and adolescents (Fig. 1). Figure 1 The most suitable way for children and teenagers to lose weight is considered by the public.

### Perceptions and attitudes of respondents towards MBS for weight loss in children and adolescents

**Fig. 2:**
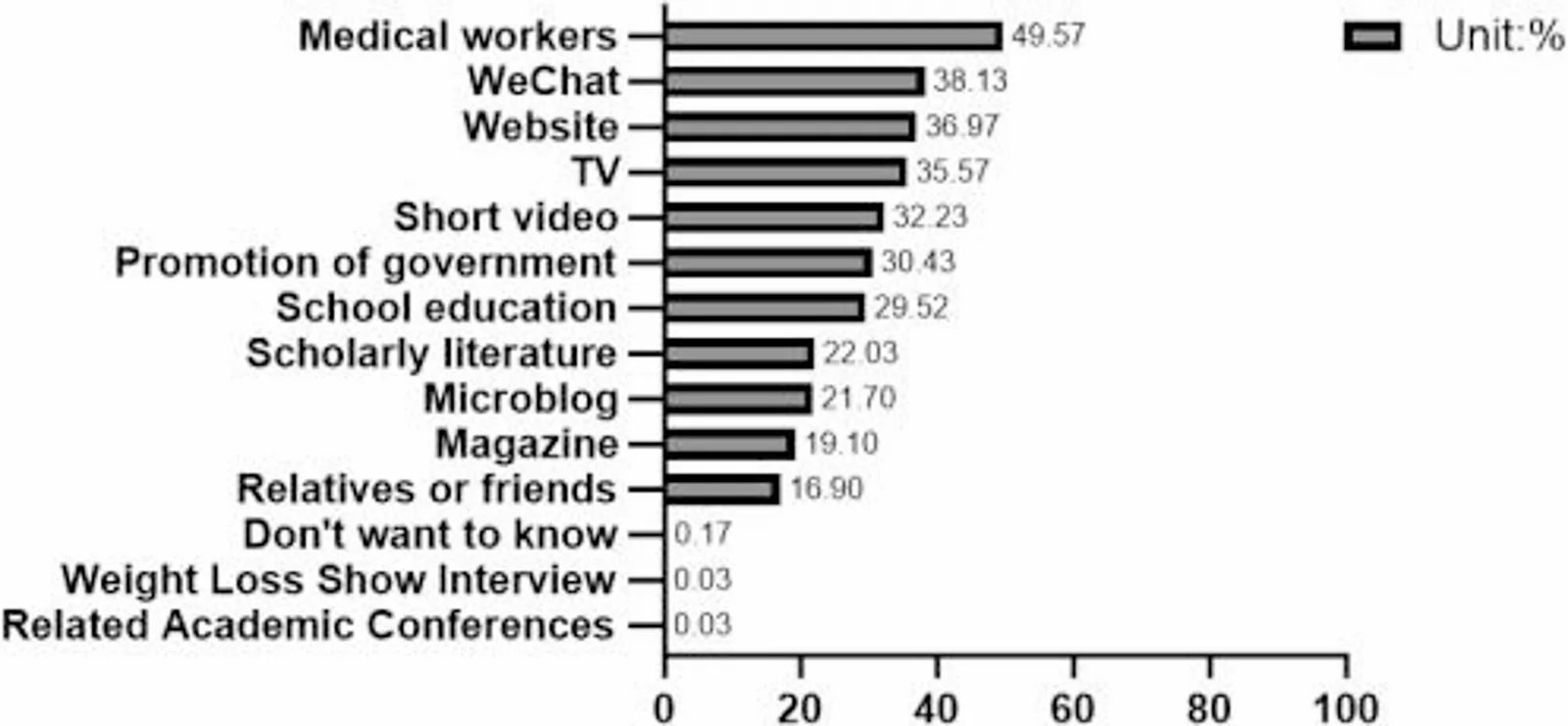
The public wants the best access to MBS information

47.53% of the residents had heard about treating obesity and metabolic diseases through bariatric surgery before this survey. Most of them learned about it through websites (49.96%) and television (49.96%), while school education (8.14%), government propaganda (4.75%), self-education (0.16%), and hospital propaganda (0.16%) were the less frequently mentioned channels (eFigure 2). The largest number of people (49.57%) indicated that they wanted to learn about bariatric surgery from healthcare professionals (Fig. 2). Figure 2 The public wants the best access to MBS information, and 30.43% wanted to get information from government propaganda materials, which is a large difference from the percentage of people who got information from government propaganda (4.75%).

Regarding the perception of “the role of MBS in children and adolescents”, the results showed that the scores of respondents’ perceptions of the role of bariatric surgery for children and adolescents (*eTable 8*) and their willingness to have their children undergo bariatric surgery (*eTable 9*) were statistically significant in relation to gender and occupation, with females (20%) scoring higher than males (14.2%) and healthcare being the highest scoring occupation; therefore, females (61.4%) and those in healthcare (66.9%) were also more likely to have their children with overweight or obesity undergo bariatric surgery. The people with overweight and obesity people are significantly more likely to have their children undergo bariatric surgery (90.2%). The concerns of those who were not willing to have their children undergo bariatric surgery (60.96%) were mainly lack of knowledge about the procedure(eFigure 3).

We also investigated the benefits of MBS for children and adolescents according to those who could undergo the procedure (86.10%), with weight loss (84.78%), improved self-confidence/improved self-image (78.97%), and improved disease (e.g., reduced drug use) (70.3%) being relatively well known. Still, better performance in interpersonal interactions and improved academic performance mention rates were only 51.70% and 25.39%(eFigure 4). Regarding the age at which their children should undergo surgery (*eTable 10*), 40% of the respondents chose “age is not an issue for me”, 18.6% of the respondents thought that surgery at the age of 12–18 was appropriate, and respondents without children (68.2%) were more unconcerned about the age at which they underwent surgery. When the child was obesity-related at the age of 7–18 and had a strong desire to undergo surgery (*eTable 11*), 69.2% would comply with the treatment wishes of the children themselves. In comparison, respondents without children (72.8%) and those who were overweight/obesity themselves (93.5%) were more likely to opt for surgery.

Binary logistic regression analysis was used to identify factors associated with residents’ perception of obesity as a disease. The independent variables included gender, age, occupation, income, and BMI. The overall model was statistically significant (Omnibus test: χ² = 99.096, df = 26, *p* < 0.001). The classification accuracy was 81.7%, and the area under the ROC curve was 0.631 (95% CI: 0.596–0.667).Females (OR = 1.913), middle-aged and older adults (OR = 3.39–4.35), and healthcare workers(OR = 1.988) showed higher recognition of “obesity as a disease,” whereas income level and self-reported BMI were not significantly associated with this perception.(Table 4).

Variables that showed significant differences in univariate analysis, along with the variable “whether obesity is considered a disease,” were entered as independent variables in a binary logistic regression analysis to identify factors associated with supportive attitudes toward bariatric surgery for children and adolescents. The overall model was statistically significant (Omnibus test: χ² = 138.509, df = 27, *p* < 0.001). The classification accuracy of the model was 60.4%, and the area under the ROC curve was 0.622 (95% CI: 0.602–0.642).The results showed that females were more supportive than males (OR = 1.301, 95% CI: 1.104–1.533, *p* = 0.002). Significant differences were also found across age groups (*p* < 0.001) and occupational groups (*p* = 0.003),respondents who held the view that “obesity is a disease for children and adolescents” showed higher supportive attitudes (OR = 0.650, 95% CI: 0.536–0.788, *p* < 0.001).(Table 5).

**Table 4:** Binary logistic regression analysis of factors associated with respondents’ perception that “obesity in children and adolescents is a disease”

Variable	OR	95% CI	*P* value
Gender (ref: Male)			
Female	1.913	(1.555, 2.353)	< 0.001
Age (ref: <18 years)			0.007
18–29 years	3.392	(1.749, 6.578)	< 0.001
30–39 years	3.606	(1.794, 7.248)	< 0.001
40–49 years	3.683	(1.768, 7.673)	< 0.001
≥ 50 years	4.346	(1.803, 10.476)	0.001
Occupation (ref: Finance)			0.024
Healthcare	1.988	(1.242, 3.181)	0.004

Note: *OR *odds ratio, *CI *confidence interval. Dependent variable coding: 1 = yes, 0 = no/not sure

**Table 5:** Binary logistic regression analysis of factors associated with willingness to support MBS surgery among children and adolescents

Variable	OR	95% CI	*p* value
Sex (ref: Male)			
Female	1.301	(1.104, 1.533)	0.002
Age			< 0.001
Occupation (ref: Finance industry)			0.003
Healthcare industry	1.515	(1.095, 2.095)	0.012
Freelancer	1.509	(1.008, 2.258)	0.046
Student	1.578	(1.071, 2.325)	0.021
Perceive obesity as a disease (ref: Yes)			
No	0.650	(0.536, 0.788)	< 0.001

*Note: *OR *odds ratio, *CI *confidence interval. Dependent variable codingcoded as 1 = support, 0 = non-support

## Discussion

The World Health Organization (WHO) estimated that at least one in five children worldwide is overweight, and 41 million children are in obesity [19, 20]. According to our data, the number of children with overweight or obesity has risen since 2015 [1]. In the United States, 19.3% of children (aged 2 to 19 years) were in obesity in 2018, with 6.1% in severe obesity and another 16.1% overweight [21]. During the 2012–2013 academic year, 33% of UK sixth-grade students (aged 10–11 years) were overweight or obesity [22]. This suggests that the increase in overweight and obesity among children and adolescents is lower in the regions surveyed in this study than in the other areas. The BMI of children and adolescents was related to their birth weight and the BMI of their parents, which is the same as the results of previous studies [23–25].

Our study shows that, males with lower education, older age and lower annual household income needed to be more knowledgeable about BMI. Females could assess their children’s body size more accurately than males. The older the children, the more likely their parents were to overestimate their body size class, similar to the results of foreign studies [22]. If parents misjudge their children’s weight, they will be unable to make the best interventions for their children, so we need to develop specific programs for obesity-related health education for different populations.

In our study, for the three questions—“factors causing obesity in children and adolescents,” “complications caused by obesity,” and “the role of MBS in children and adolescents”—each question is a multiple-response item with multiple options. To facilitate statistical analysis, we assigned scores to the responses for these three questions. Each option selected by a participant was awarded one point. 45% of the respondents could correctly and completely identify the factors contributing to obesity in children and adolescents, including genetic, behavioral (diet and physical activity), psychological, and environmental factors [24]. It is worth mentioning that childhood is a crucial period for forming behaviors related to eating and physical activity and that various environmental factors influence the development of good habits [26, 27].

Our data indicates that participants have incomplete knowledge about obesity-related complications and need to popularize more obesity-related diseases; obesity not only causes harm to children and adolescents’ bodies, but also affects their mental health, personality and social skills. Some studies have shown that obesity has a negative impact on adolescents’ academic performance in China, in addition, overweight/obesity is associated with poorer functional performance on campus, social skills and reduced participation in school activities [28, 29]. Adolescents with overweight/obesity have lower scores than normal adolescents regarding relationships with teachers and other students, participation in school activities, and overall mood toward school [30]. Such adolescents are more likely to have negative psychological conditions at school, especially girls with overweight/obesity, possibly because they face more weight-related stigma than boys and may be more vulnerable to teasing or bullying [31–33].

Therefore, we should actively pay attention to the negative effects of overweight/obesity on children and adolescents in all aspects, include the harm of various complications at the physical level, the negative psychological aspects such as low self-esteem caused by discrimination and ridicule from the outside world, and whether children and adolescents have experienced unfair treatment in terms of education, resource allocation and merit evaluation in personal development, etc.

In most countries, the prevalence of overweight in children is higher than that of obesity [20]. Children who are overweight at age 5 are four times more likely to be in obesity at ages 5–14 than children of normal weight [34]. Overweight children are more likely than normal-weight or children in obesity to change their BMI classification at the one-year follow-up [35]. Therefore, childhood obesity prevention should target overweight children who are at high risk of obesity but are not yet in obesity, which may be more effective than treating children with obesity; identifying risk factors and intervening in overweight children at high risk for obesity is an effective strategy to prevent childhood obesity; and, childhood weight management strategies should begin at the earliest possible age [36]. With the relative intellectual and psychological immaturity of children and adolescents, managing obesity in these groups can be difficult. Only a combination of school life, family activities, environmental and cultural practices, and management approaches supported by parents and teachers are promising policies and strategies.

The survey found that respondents lacked sufficient understanding of MBS, which is underutilized. According to one study, only 0.04% of adolescents with severe obesity meet the indications for MBS in the United States receive MBS each year [37]. The most suitable age for surgery is 12–18 years old for 18% of the respondents, 19–29 years old for 4.6%, and age is not an issue for 40%. Regarding the timing of surgery, the American Academy of Pediatrics [37] mentioned that MBS should be adopted regardless of age as long as the operation requirements are met [34].

Age was positively correlated with the recognition that “obesity is a disease.” Middle-aged and older adults may have more direct experience of the health hazards associated with obesity.The higher recognition that “obesity is a disease” and higher support rate for surgery among females may be because women typically bear more responsibility for family health care, are more sensitive to children’s health issues, or are more concerned about the impact of obesity on their children’s future quality of life. The higher support rate among certain occupational groups (e.g., healthcare workers, educators, white-collar professionals) may be related to their level of knowledge about medical interventions, health literacy, or socioeconomic status. Considering obesity as a disease implies accepting that it requires medical intervention, and bariatric surgery naturally becomes one of the options to be considered.

As in adults, MBS in children and adolescents is equally effective in reducing weight and alleviating complications [21, 38]. The average weight loss in children and adolescents after sleeve gastrectomy (SG) is 14.1 kg/m2, and the average weight loss after Roux-en-Y gastric bypass (RYGB) is 16.6 kg/m2 [39]. The average weight loss remains 13.1–17.0 kg/m2 5–8 years after RYGB, with 95% of patients experiencing remission of type 2 diabetes, 86% of renal abnormalities, 74% of hypertension, and 66% of dyslipidemia, patients also showed significant improvement in quality of life [14]. Accepting MBS not only resulted in sustained weight loss, improved obesity-related comorbidities and weight-related quality of life [40], the improvement in depression, anxiety, cognitive and executive functioning, and self-image after MBS in adolescents was also observed [41].

In addition, when the indications for MBS are met [42], Our data indicate that children’s treatment wishes also influence the parents’ medical decisions.Respondents were concerned about postoperative complications or adverse effects like malnutrition, impact on puberty development…etc, which are common postoperative complications [43]. One study showed that the rate of complications during postoperative hospitalization decreased from 8.8% in 2005 to 2.0% in 2014 [44], with more pronounced iron deficiency anemia and vitamin B12 deficiency observed in long-term follow-up, with 46% of patients suffering from anemia and 78% reporting low vitamin D levels three years after surgery [45–48]. Another study comparing outcomes in adolescents (14–18 years old) and adults (25–50 years old) who underwent RYGB found similar weight loss in both groups (26% vs. 29%). Still, significantly higher rates of T2D remission (86% vs. 53%) and hypertension remission (68% vs. 41%) in the adolescent group at five years postoperatively [49]. This is particularly important because the youth-onset T2D subtype is more aggressive than the adult-onset T2D subtype [50, 51]. Early intervention can save potential islet function, prevent irreversible β-cell damage and prevent vascular remodelling and consequent atherosclerosis [50, 52, 53]. The results of the study also showed a greater reduction in BMI and weight in adolescents compared to adults at 4 years postoperatively [50, 52–54]. This evidence emphasizes the importance of early MBS for children and adolescents with obesity or severe complications [55].

Several studies have shown that the quality and content of information about MBS on the Internet is usually poor [56, 57]. Therefore, when we surveyed residents about the sources they would like to learn about MBS, the majority wanted to know about it from healthcare professionals (49.57%), probably because they felt that the information they received was more authoritative. Some studies have shown that people with obesity who have received services from a weight management medical team are more likely to accept MBS because these patients may be better informed about the benefits of surgery and the risks of severe obesity [58]. Most children and adolescents are still in school, so students’ knowledge of MBS and obesity can be enhanced through health education in schools. It is worth noting that 30.43% of people want to get information from government promotion materials, which is a big difference from the percentage of people who get information from government propaganda (4.75%). Therefore, to popularize the knowledge related to weight loss and MBS, we can adopt medical and nursing staff combined with various media ways to increase promotion, such as short videos and tweets released by official hospital accounts. And relevant government departments should pay more attention to the publicity of MBS to increase the public awareness rate and provide authoritative information support for people with related medical service needs [59, 60]. Weight bias and obesity stigma also hinder people from receiving treatments, which should raise our concerns [61]. Implementation of artificial intelligence such as large language models (LLMs) is also a novel way for health education [62].

### Strengths and limitations

To the best of our knowledge, this is the first national survey on the perceptions and attitudes of a subset of Chinese residents towards obesity and MBS for weight loss in children and adolescents. The questionnaire data collection is comprehensive, including general data from adults and adolescents, and a subset of Chinese residents' perceptions and attitudes towards adolescent obesity, weight loss and MBS, which further enriches the relevant information and evidence at home and abroad and provides a basis for improving the utilization rate of MBS for children and adolescents with obesity. However, the present study also has some limitations that should be taken into account when interpreting our results. The present study actually employed an online convenience sampling method. This sampling strategy introduced potential biases in several aspects, significantly limiting the representativeness, generalizability, and external validity of the research findings. A detailed explanation is provided below.

1. Selection Bias. Online convenience sampling relies on participants voluntarily clicking on links, scanning QR codes, or actively engaging in the study through specific platforms (e.g., social media, online communities, survey crowdsourcing platforms). This non-probability sampling approach means that the composition of the sample is not based on random principles but is instead constrained by factors such as participant accessibility, interest, time, and motivation. Consequently, the study sample may exhibit systematic differences from non-participants on key variables (e.g., health literacy, internet usage habits, social attitudes, economic status). This selection bias implies that any effects or associations observed in this study may be either overestimated or underestimated and cannot be simply generalized to the entire Chinese population.
2. Overrepresentation of Highly Educated Participants. Approximately 87.7% of the participants in this study's sample hold a bachelor's degree or higher. In contrast, according to data from the Seventh National Population Census (2020) released by the National Bureau of Statistics of China, only about 15% of the Chinese population has an associate degree or higher (specifically around 15.5%, with an even lower proportion holding a bachelor's degree or above)[63].This substantial discrepancy clearly indicates that the study's sample is severely biased towards highly educated individuals. This overrepresentation may have the following specific impacts on the research findings: Cognitive and Behavioral Patterns: Highly educated individuals often differ significantly from those with lower educational attainment in areas such as information acquisition, health behaviors, policy understanding, and risk perception. For example, they may find it easier to comprehend complex questions in a questionnaire or may hold particular inclinations regarding certain social issues. Therefore, the conclusions of this study may only apply to highly educated groups and fail to reflect the realities of individuals with low or intermediate levels of education. Confounding with Socioeconomic Status: Educational level is often highly correlated with income, occupation type, place of residence (urban/rural), and access to healthcare. The overrepresentation of highly educated participants may inadvertently amplify the characteristics of the urban middle class and above while marginalizing key groups such as the rural population, low-skilled workers, and the elderly.
3. Exclusion of Individuals Without Internet Access. This study was conducted entirely online, thereby excluding all individuals who lack access to the internet or stable internet usage conditions. These non-internet users are primarily concentrated in the following groups: Rural residents, especially those in remote areas; Elderly individuals (aged 60 and above);Individuals with low educational attainment; Low-income or disabled populations. Because these groups exhibit systematic differences in key demographic characteristics compared to Chinese internet users, the sample in this study effectively represents only a subset (and a convenience subset) of the "internet-using population of China," rather than the "total population of China." Consequently, any frequency estimates derived from this study (e.g., the prevalence of a certain behavior, the proportion holding a certain attitude) should not be directly interpreted as national-level estimates.
4. . Summary of Impact on Research Findings and Their Generalizability. Synthesizing the sources of bias described above, the conclusions of this study should be generalized to the following populations or situations only with extreme caution: Populations with lower educational levels (e.g., those with middle school education or less);Populations without internet access or with limited digital engagement (e.g., rural elderly, some low-income groups);Populations with low response rates to online surveys (e.g., those uninterested in research, time-constrained, or with low trust levels).
5. Weight and height were filled in as self-reports, so errors may occur and affect the association between individual factors.
6. Our findings represent a baseline view from 2022. We state that the perceptions and attitudes we captured likely reflect a specific moment in time—at the tail end of the COVID-19 pandemic in China—and may not account for subsequent, rapid societal shifts, such as the recent surge in popularity of GLP-1 receptor agonists.

## Conclusions

A subset of Chinese residents are not fully aware of the harm of obesity among children and adolescents and its related complications; their attitude toward weight reduction and MBS for children and adolescents is relatively conservative, mainly because they do not know enough about the surgery and underestimate the harm of obesity in children and adolescents and the positive effects that the surgery can bring. We should join hands with hospitals, schools, communities and policy makers to focus on the physical and mental health of children and adolescents with overweight and obesity and strengthen health education on weight management and obesity prevention to regulate personal behavior better and promote public health to create a positive health culture. Objectively publicize the requirements, effects and complications of MBS in multiple ways so that children and adolescents in need and their parents can carefully choose treatment in the context of the benefits and risks associated with surgery. 

## Supplementary Information

Supplementary Material 1.

Supplementary Material 2.

## Data Availability

The dataset(s) supporting the conclusions of this article is(are) included within the article (and its additional file(s)).

## Abbreviations

MBS: Metabolic and bariatric surgery
BMI: Body mass index
CNY: Chinese Yuan
WHO: The World Health Organization
SG: Sleeve gastrectomy
RYGB: Roux-en-Y gastric bypass

## References

[1] Lobstein T, Jackson-Leach R. Planning for the worst: estimates of obesity and comorbidities in school-age children in 2025. Pediatr Obes. 2016;11(5):321–5. 10.1111/ijpo.12185 27684716

[2] Ma SJ, et al. [Analysis on the trend of overweight and obesity of children and adolescents in 9 provinces of China from 1991 to 2015]. Zhonghua Yu Fang Yi Xue Za Zhi. 2020;54(2):133–8. 10.3760/cma.j.issn.0253-9624.2020.02.004 32074698

[3] Gao L, et al. A 3-year longitudinal study of effects of parental perception of children’s ideal body image on child weight change: The Childhood Obesity Study in China mega-cities. Prev Med. 2020;132:105971. 10.1016/j.ypmed.2019.105971 PMC7024657 31899255

[4] Simmonds M, et al. Predicting adult obesity from childhood obesity: a systematic review and meta-analysis. Obes Rev. 2016;17(2):95–107. 10.1111/obr.12334 26696565

[5] Worldwide trends in. body-mass index, underweight, overweight, and obesity from 1975 to 2016: a pooled analysis of 2416 population-based measurement studies in 128.9 million children, adolescents, and adults. Lancet. 2017;390(10113):2627–42. 10.1016/S0140-6736(17)32129-3 PMC5735219 29029897

[6] Hales CM, et al. Trends in Obesity and Severe Obesity Prevalence in US Youth and Adults by Sex and Age, 2007–2008 to 2015–2016. JAMA. 2018;319(16):1723–5. 10.1001/jama.2018.3060 PMC5876828 29570750

[7] Mittal M, Jain V. Management of Obesity and Its Complications in Children and Adolescents. Indian J Pediatr. 2021;88(12):1222–34. 10.1007/s12098-021-03913-3 PMC8491444 34609654

[8] Lauby-Secretan B, et al. Body Fatness and Cancer–Viewpoint of the IARC Working Group. N Engl J Med. 2016;375(8):794–8. 10.1056/NEJMsr1606602 PMC6754861 27557308

[9] Shamriz O, et al. Higher body mass index in 16–19 year-old Jewish Adolescents of North African, Middle Eastern and European Origins is a Predictor of Acute Myeloid Leukemia: a cohort of 2.3 million Israelis. Cancer Causes Control. 2017;28(4):331–9. 10.1007/s10552-017-0863-5 28258513

[10] Berenson GS. Health consequences of obesity. Pediatr Blood Cancer. 2012;58(1):117–21. 10.1002/pbc.23373 22076834

[11] Bussler S, et al. Novel Insights in the Metabolic Syndrome in Childhood and Adolescence. Horm Res Paediatr. 2017;88(3–4):181–93. 10.1159/000479510 28848168

[12] Mahawar KK, et al. The first modified Delphi consensus statement on sleeve gastrectomy. Surg Endosc. 2021;35(12):7027–33. 10.1007/s00464-020-08216-w 33433676

[13] Ren Y, et al. Effect of Roux-en-Y gastric bypass with different pouch size in Chinese T2DM patients with BMI 30–35 kg/m2. Obes Surg. 2015;25(3):457–63. 10.1007/s11695-014-1411-y 25236397

[14] Inge TH, et al. Weight Loss and Health Status 3 Years after Bariatric Surgery in Adolescents. N Engl J Med. 2016;374(2):113–23. 10.1056/NEJMoa1506699 PMC4810437 26544725

[15] Kelly AS, et al. Severe obesity in children and adolescents: identification, associated health risks, and treatment approaches: a scientific statement from the American Heart Association. Circulation. 2013;128(15):1689–712. 10.1161/CIR.0b013e3182a5cfb3 24016455

[16] Diao X, et al. Knowledge and Attitudes Towards Obesity and Bariatric Surgery in University Students: a National Survey. Obes Surg. 2022;32(9):2869–79. 10.1007/s11695-022-06157-y 35729447

[17] Singh UD, Chernoguz A. Parental attitudes toward bariatric surgery in adolescents with obesity. Surg Obes Relat Dis. 2020;16(3):406–13. 10.1016/j.soard.2019.12.010 PMC7832083 31917199

[18] Eysenbach G. Improving the quality of web surveys: the checklist for reporting results of internet E-surveys (CHERRIES). J Med Internet Res. 2004;6(3):1–6. 10.2196/jmir.6.3.e34 PMC1550605 15471760

[19] Zong XN, Li H. Construction of a new growth references for China based on urban Chinese children: comparison with the WHO growth standards. PLoS ONE. 2013;8(3):e59569. 10.1371/journal.pone.0059569 PMC3602372 23527219

[20] Ng M, et al. Global, regional, and national prevalence of overweight and obesity in children and adults during 1980–2013: a systematic analysis for the Global Burden of Disease Study 2013. Lancet. 2014;384(9945):766–81. 10.1016/S0140-6736(14)60460-8 PMC4624264 24880830

[21] Salimi-Jazi F, et al. Trends in Adolescent Bariatric Procedures: a 15-Year Analysis of the National Inpatient Survey. Obes Surg. 2022;32(11):3658–65. 10.1007/s11695-022-06265-9 36103080

[22] Black JA, et al. Child obesity cut-offs as derived from parental perceptions: cross-sectional questionnaire. Br J Gen Pract. 2015;65(633):e234–9. 10.3399/bjgp15X684385 PMC4377621 25824183

[23] Whitaker RC, et al. Predicting obesity in young adulthood from childhood and parental obesity. N Engl J Med. 1997;337(13):869–73. 10.1056/NEJM199709253371301 9302300

[24] Lee EY, Yoon KH. Epidemic obesity in children and adolescents: risk factors and prevention. Front Med. 2018;12(6):658–66. 10.1007/s11684-018-0640-1 30280308

[25] Vasylyeva TL, et al. Obesity in prematurely born children and adolescents: follow up in pediatric clinic. Nutr J. 2013;12(1):150. 10.1186/1475-2891-12-150 PMC3842808 24252330

[26] Karnik S, Kanekar A. Childhood obesity: a global public health crisis. Int J Prev Med. 2012;3(1):1–7. PMC3278864 22506094

[27] Grundy SM. Multifactorial causation of obesity: implications for prevention. Am J Clin Nutr. 1998;67(3 Suppl):S563–72. 10.1093/ajcn/67.3.563S 9497171

[28] Wu N, et al. Childhood Obesity and Academic Performance: The Role of Working Memory. Front Psychol. 2017;8:611. 10.3389/fpsyg.2017.00611 PMC5395561 28469593

[29] Ma L, et al. Overweight and Obesity Impair Academic Performance in Adolescence: A National Cohort Study of 10,279 Adolescents in China. Obes (Silver Spring). 2020;28(7):1301–9. 10.1002/oby.22801 32447840

[30] Veldwijk J, et al. Overweight and school performance among primary school children: the PIAMA birth cohort study. Obes (Silver Spring). 2012;20(3):590–6. 10.1038/oby.2011.327 22030985

[31] Tang-Peronard JL, Heitmann BL. Stigmatization of obese children and adolescents, the importance of gender. Obes Rev. 2008;9(6):522–34. 10.1111/j.1467-789X.2008.00509.x 18643945

[32] Puhl RM, Luedicke J. Weight-based victimization among adolescents in the school setting: emotional reactions and coping behaviors. J Youth Adolesc. 2012;41(1):27–40. 10.1007/s10964-011-9713-z 21918904

[33] Sagar R, Gupta T. Psychological Aspects of Obesity in Children and Adolescents. Indian J Pediatr. 2018;85(7):554–9. 10.1007/s12098-017-2539-2 29150753

[34] Cunningham SA, Kramer MR, Narayan KM. Incidence of childhood obesity in the United States. N Engl J Med. 2014;370(5):403–11. 10.1056/NEJMoa1309753 PMC4017620 24476431

[35] Lee EY, et al. Study Time after School and Habitual Eating Are Associated with Risk for Obesity among Overweight Korean Children: A Prospective Study. Obes Facts. 2018;11(1):46–55. 10.1159/000486132 PMC5869488 29408816

[36] Serdula MK, et al. Do obese children become obese adults? A review of the literature. Prev Med. 1993;22(2):167–77. 10.1006/pmed.1993.1014 8483856

[37] Griggs CL, et al. National Trends in the Use of Metabolic and Bariatric Surgery Among Pediatric Patients With Severe Obesity. JAMA Pediatr. 2018;172(12):1191–2. 10.1001/jamapediatrics.2018.3030 PMC6279469 30357351

[38] Pratt J, et al. ASMBS pediatric metabolic and bariatric surgery guidelines, 2018. Surg Obes Relat Dis. 2018;14(7):882–901. 10.1016/j.soard.2018.03.019 PMC6097871 30077361

[39] Olbers T, et al. Two-year outcome of laparoscopic Roux-en-Y gastric bypass in adolescents with severe obesity: results from a Swedish Nationwide Study (AMOS). Int J Obes (Lond). 2012;36(11):1388–95. 10.1038/ijo.2012.160 23007037

[40] Thenappan A, Nadler E. Bariatric Surgery in Children: Indications, Types, and Outcomes. Curr Gastroenterol Rep. 2019;21(6):24. 10.1007/s11894-019-0691-8 31025124

[41] Mackey ER, et al. Cognitive Performance as Predictor and Outcome of Adolescent Bariatric Surgery: A Nonrandomized Pilot Study. J Pediatr Psychol. 2018;43(8):916–27. 10.1093/jpepsy/jsy028 PMC6093427 29788390

[42] Yang W, Wang C. Metabolic Surgery Needs Stronger Endorsement in Asian T2DM Patients with Low BMI. Obes Surg. 2022;32(1):212–3. 10.1007/s11695-021-05636-y 34342801

[43] Zhang W, et al. Hair Loss After Metabolic and Bariatric Surgery: a Systematic Review and Meta-analysis. Obes Surg. 2021;31(6):2649–59. 10.1007/s11695-021-05311-2 PMC8113177 33675022

[44] Zhang W, et al. Importance of Maintaining Zinc and Copper Supplement Dosage Ratio After Metabolic and Bariatric Surgery. Obes Surg. 2021;31(7):3339–40. 10.1007/s11695-021-05379-w 33782849

[45] Guan B, et al. Nutritional Deficiencies in Chinese Patients Undergoing Gastric Bypass and Sleeve Gastrectomy: Prevalence and Predictors. Obes Surg. 2018;28(9):2727–36. 10.1007/s11695-018-3225-9 29754386

[46] Wang C, et al. Prevalence of electrolyte and nutritional deficiencies in Chinese bariatric surgery candidates. Surg Obes Relat Dis. 2016;12(3):629–34. 10.1016/j.soard.2015.12.009 27012874

[47] Yang W, et al. Global variations in preoperative practices concerning patients seeking primary bariatric and metabolic surgery (PACT Study): A survey of 634 bariatric healthcare professionals. Int J Obes (Lond). 2022;46(7):1341–50. 10.1038/s41366-022-01119-x 35399108

[48] Alqahtani AR, et al. Ten-Year Outcomes of Children and Adolescents Who Underwent Sleeve Gastrectomy: Weight Loss, Comorbidity Resolution, Adverse Events, and Growth Velocity. J Am Coll Surg. 2021;233(6):657–64. 10.1016/j.jamcollsurg.2021.08.678 34563670

[49] Inge TH, et al. Five-Year Outcomes of Gastric Bypass in Adolescents as Compared with Adults. N Engl J Med. 2019;380(22):2136–45. 10.1056/NEJMoa1813909 PMC7345847 31116917

[50] Beamish AJ, D’Alessio DA, Inge TH. Controversial Issues: When the drugs don’t work, can surgery provide a different outcome for diabetic adolescents? Surg Obes Relat Dis. 2015;11(4):946–8. 10.1016/j.soard.2015.03.006 25980332

[51] Shah AS, et al. Metabolic outcomes of surgery in youth with type 2 diabetes. Semin Pediatr Surg. 2020;29(1):150893. 10.1016/j.sempedsurg.2020.150893 PMC7125189 32238292

[52] Beamish AJ, Olbers T. Metabolic and bariatric surgery in adolescents. Nat Rev Gastroenterol Hepatol. 2019;16(10):585–7. 10.1038/s41575-019-0187-x 31308496

[53] Gunnell DJ, et al. Childhood obesity and adult cardiovascular mortality: a 57-y follow-up study based on the Boyd Orr cohort. Am J Clin Nutr. 1998;67(6):1111–8. 10.1093/ajcn/67.6.1111 9625081

[54] Stanford FC, Mushannen T, Cortez P, et al. Comparison of Short and Long-Term Outcomes of Metabolic and Bariatric Surgery in Adolescents and Adults. Front Endocrinol (Lausanne). 2020;11:157. Published 2020 Mar 24. 10.3389/fendo.2020.00157. PMC7105703 32265846

[55] Yang W, Wang C. Long-Term Complications in Youth-Onset Type 2 Diabetes. N Engl J Med. 2021;385(21):2014–5. 10.1056/NEJMc2114053 34788518

[56] Barajas-Gamboa JS, et al. Quality of Information About Bariatric Surgery on the Internet: A Two-Continent Comparison of Website Content. Obes Surg. 2020;30(5):1736–44. 10.1007/s11695-019-04375-5 31950316

[57] Daraz L, et al. Can Patients Trust Online Health Information? A Meta-narrative Systematic Review Addressing the Quality of Health Information on the Internet. J Gen Intern Med. 2019;34(9):1884–91. 10.1007/s11606-019-05109-0 PMC6712138 31228051

[58] Arhi C, et al. The Effect of Participation in Tier 3 Services on the Uptake of Bariatric Surgery. Obes Surg. 2021;31(6):2529–36. 10.1007/s11695-021-05303-2 PMC7962433 33725296

[59] Zhang W, et al. Perceptions and Attitudes Toward Obesity and its Management in Migrants and Rural Residents in China: a Cross-sectional Pilot Study. Obes Surg. 2022;32(1):152–9. 10.1007/s11695-021-05755-6 34643851

[60] Fan M, et al. Knowledge and Attitudes Towards Obesity and Bariatric Surgery in Chinese Nurses. Obes Surg. 2020;30(2):618–29. 10.1007/s11695-019-04173-z 31758470

[61] Zhang W, et al. Ending Obesity Stigma and Discrimination: Starting From Healthcare Professionals. Am J Gastroenterol. 2021;116(8):1753. 10.14309/ajg.0000000000001269 33878044

[62] Au K, Yang W. Auxiliary use of ChatGPT in surgical diagnosis and treatment. Int J Surg. 2023;109(12):3940–3. PMID: 37678271. 10.1097/JS9.0000000000000686. PMC10720849 37678271

[63] National Bureau of Statistics of China, Office of the Leading Group of the State Council for the Seventh National Population Census. Bulletin of the seventh national population census (No. 6) – Educational attainment of the population . 2021 [cited 2026 May 16].Available from: http://www.stats.gov.cn/english/PressRelease/202105/t20210513_1817386.html.

