# Supplementary material for: Perceptions and attitudes towards obesity and metabolic and bariatric surgery for weight loss in children and adolescents: a survey among a subset of Chinese residents
Source: BMC Public Health. 2026 Jul 18;26:2958. doi: 10.1186/s12889-026-28495-7 (PMC13663007; doi:10.1186/s12889-026-28495-7)
Supplement: Supplementary file 1 [file 12889_2026_28495_MOESM2_ESM.docx]

eTables in Supplementary Files

eTable 1. Different parents' evaluation of their children's body shape ---------------------------------- page2

eTable 2. Public' awareness of BMI definition--------------------------------------------------------------page4

eTable 3.Public' awareness of calculate BMI----------------------------------------------------------------page6

eTable 4. Public recognition of childhood and adolescent obesity as a disease-------------------------page8

eTable 5. Public perception of the causes of obesity in children and adolescents--------------------page10

eTable 6. Public awareness of the dangers of obesity in children and adolescents-------------------page12

eTable 7. Public perception of the risks of obesity in children, adolescents and adults--------------page14

eTable 8. Public' awareness of the benefits of MBS------------------------------------------------------page16

eTable 9. Public' approval of their child's MBS-----------------------------------------------------------page18

eTable 10.Age at which public consider children and adolescents suitable for MBS----------------page20

eTable 11. Public' approval of their child's desire to have MBS----------------------------------------page21

eTable 1 Different parents' evaluation of their children's body shape

| Variables | Parents' evaluation of their children' s body size | | | P value | Coefficient of association |
| --- | --- | --- | --- | --- | --- |
|  | Smaller than actual | Same as actual | Larger than actual |  |  |
| Gender of parents | | |  | 0.00 | 0.4 |
| Male | 179(43.2) | 198(47.8) | 37(8.9) |  |  |
| Female | 184(34.3) | 304(56.6) | 49(9.1) |  |  |
| Parents' education | | |  | 0.25 | 0.01 |
| Junior high school and below | 12(35.5) | 21(61.8) | 1(2.9) |  |  |
| High school or junior college | 29(29.9) | 58(59.8) | 10(10.3) |  |  |
| Undergraduate or college | 255(40.7) | 314(50.1) | 58(9.3) |  |  |
| Master's degree and above | 67(34.7) | 109(56.6) | 17(8.8) |  |  |
| Parents' age(years) | | |  | 0.001 | 0.129* |
| <30 | 25(48.1) | 23(44.2) | 4(7.7) |  |  |
| 30-40 | 266(40.7) | 341(52.1) | 47(7.2) |  |  |
| 41-50 | 67(28.9) | 133(57.3) | 32(13.8) |  |  |
| >50 | 5(38.5) | 5(38.5) | 3(23.1) |  |  |
| Age of children(years) | | | | 0.00 | 0.165* |
| 2-6 | 166(46.8) | 164(46.2) | 25(7.0) |  |  |
| 7-12 | 146(36.8) | 218(54.9) | 33(8.3) |  |  |
| 12-18 | 51(25.6) | 120(60.3) | 28(14.1) |  |  |
| Sex of children | | | | 0.804 | 0.01 |
| Male | 200(38.8) | 272(52.7) | 44(8.5) |  |  |
| Female | 163(37.5) | 230(52.9) | 42(9.7) |  |  |
| Careers | | |  | 0.621 | 0.166* |
| Finance | 44(39.6) | 56(50.5) | 11(9.9) |  |  |
| Education | 51(33.8) | 85(56.3) | 15(9.9) |  |  |
| Health Care | 41(35.3) | 69(59.5) | 6(5.2) |  |  |
| Media,Arts | 4(25) | 10(62.5) | 2(12.5) |  |  |
| Industrial Technology Related | 39(44.8) | 42(48.3) | 6(6.9) |  |  |
| Science,information technology related | 30(42.9) | 36(51.4) | 4(5.7) |  |  |
| Agriculture,forestry,fishery and water production | 10(37) | 15(55.6) | 2(7.4) |  |  |
| Service industry | 53(43.4) | 59(48.4) | 10(8.2) |  |  |
| Transportation equipment related | 5(38.5) | 5(38.5) | 3(23.1) |  |  |
| Self-employed | 28(36.4) | 41(53.2) | 8(10.4) |  |  |
| Military related | 1(50.0) | 0(0.0) | 1(50.0) |  |  |
| Student | 0(0.0) | 1(100.0) | 0(0.0) |  |  |
| Unemployed | 6(42.9) | 8(57.1) | 0(0.0) |  |  |
| Retired | 2(66.7) | 1(33.3) | 0(0.0) |  |  |
| Full-time Mom/Dad | 27(33.8) | 45(56.2) | 8(10.0) |  |  |
| Other | 22(36.1) | 29(47.5) | 10(16.4) |  |  |
| Monthly per capita family income(yuan) | | |  | 0.200 | 0.082* |
| <3000 | 12(48.0) | 12(48.0) | 1(4.0) |  |  |
| 3000-6000 | 56(43.4) | 66(51.2) | 7(5.4) |  |  |
| 6001-9000 | 72(42.9) | 79(47.0) | 17(10.1) |  |  |
| >9000 | 223(35.5) | 345(54.8) | 61(9.7) |  |  |
| BMI of parents(kg/m^2^) | | |  | 0.181 | -0.074* |
| <18.5 | 1(29.3) | 37(63.8) | 4(6.9) |  |  |
| 18.5-23.9 | 206(36.4) | 306(54.1) | 54(9.5) |  |  |
| 24-27.9 | 101(40.6) | 126(50.6) | 22(8.8) |  |  |
| ≥28 | 39(50) | 33(42.3) | 6(7.7) |  |  |
| Parents' preference for their own body size evaluation | | | | 0.823 | 0.012* |
| Smaller | 51(37.5) | 71(52.2) | 14(10.3) |  |  |
| Normal | 227(38.1) | 320(53.7) | 49(8.2) |  |  |
| Large | 85(38.8) | 111(50.7) | 23(10.5) |  |  |

eTable 2 Do you know the body mass index(BMI)?

| Variables | Do you know the BMI | | P value | Coefficient of association |
| --- | --- | --- | --- | --- |
|  | Yes | No |  |  |
| Gender | | | 0.000 | 0.089 |
| Male | 719(55.1) | 585(44.9) |  |  |
| Female | 1095(64.0) | 616(36.0) |  |  |
| Education | | | 0.000 | 0.306 |
| Junior high school and below | 22(23.9) | 70(76.1) |  |  |
| High school or junior college | 72(25.8) | 207(74.2) |  |  |
| Undergraduate or college | 1184(59.6) | 804(40.4) |  |  |
| Master's degree and above | 536(81.5) | 122(18.5) |  |  |
| Age(years) | | | P=0.000 | 0.089 |
| <18 | 27(50.0) | 27(50.0) |  |  |
| 18-29 | 908(64.3) | 502(35.6) |  |  |
| 30-39 | 607(58.5) | 430(41.4) |  |  |
| 40-49 | 205(54.1) | 174(45.9) |  |  |
| ≥50 | 67(51.5) | 63(48.5) |  |  |
| Careers | | | 0.000 | 0.275 |
| Finance | 185(59.7) | 125(40.3) |  |  |
| Education | 297(71.7) | 117(28.2) |  |  |
| Health Care | 277(83.4) | 55(16.5) |  |  |
| Media,Arts | 76(56.7) | 58(43.2) |  |  |
| Industrial Technology Related | 128(53.8) | 110(46.2) |  |  |
| Science,information technology related | 173(65.0) | 93(34.9) |  |  |
| Agriculture,forestry,fishery and water production | 36(51.4) | 34(48.5) |  |  |
| Service industry | 169(49.1) | 175(50.8) |  |  |
| Transportation equipment related | 18(47.4) | 20(52.6) |  |  |
| Self-employed | 49(31.8) | 105(68.2) |  |  |
| Military related | 0 | 7(100.0) |  |  |
| Student | 208(73.2) | 76(26.7) |  |  |
| Unemployed | 32(41.0) | 46(58.9) |  |  |
| Retired | 16(50.0) | 16(50,0) |  |  |
| Full-time Mom/Dad | 41(41.8) | 57(58.1) |  |  |
| Other | 109(50.5) | 107(49.5) |  |  |
| Monthly per capita family income(yuan) | | | 0.000 | 0.129 |
| <3000 | 88(54.0) | 75(46.0) |  |  |
| 3000-6000 | 295(49.6) | 300(50.4) |  |  |
| 6001-9000 | 390(58.1) | 281(41.9) |  |  |
| >9000 | 1041(65.6) | 545(34.4) |  |  |
| BMI(kg/m^2^) | | | 0.068 | 0.049 |
| <18.5 | 195(58.2) | 140(41.8) |  |  |
| 18.5-23.9 | 1124(62.1) | 685(37.9) |  |  |
| 24-27.9 | 361(57.3) | 269(42.7) |  |  |
| ≥28 | 124(56.1) | 97(43.9) |  |  |

**The correlation coefficient defaults to coefficient of association*

eTable 3 Do you know how to calculate BMI?

|  | Do you know how to calculate BMI | | P value | Coefficient of association |
| --- | --- | --- | --- | --- |
| Variables | Yes | No |  |  |
| Gender | | | 0.000 | 0.071 |
| Male | 559(42.9) | 745(57.1) |  |  |
| Female | 856(50.0) | 855(50.0) |  |  |
| Education | | | 0.000 | 0.251 |
| Junior high school and below | 15(16.3) | 77(83.7) |  |  |
| High school or junior college | 59(21.3) | 218(78.7) |  |  |
| Undergraduate or college | 908(45.7) | 1080(54.3) |  |  |
| Master's degree and above | 433(65.8) | 225(34.2) |  |  |
| Age(years) | | | 0.007 | 0.069 |
| <18 | 23(42.6) | 31(57.4) |  |  |
| 18-29 | 710(50.4) | 700(49.6) |  |  |
| 30-39 | 462(44.6) | 575(55.4) |  |  |
| 40-49 | 170(44.3) | 214(55.7) |  |  |
| ≥50 | 50(38.5) | 80(61.5) |  |  |
| Careers | | | 0.000 | 0.294 |
| Finance | 134(43.2) | 176(56.8) |  |  |
| Education | 215(51.9) | 199(48.1) |  |  |
| Health Care | 268(80.7) | 64(19.3) |  |  |
| Media,Arts | 63(47.0) | 71(53.0) |  |  |
| Industrial Technology Related | 102(42.9) | 136(57.1) |  |  |
| Science,information technology related | 121(45.5) | 145(54.4) |  |  |
| Agriculture,forestry,fishery and water production | 23(32.9) | 47(67.1) |  |  |
| Service industry | 122(35.5) | 222(64.5) |  |  |
| Transportation equipment related | 16(42.1) | 22(57.9) |  |  |
| Self-employed | 38(24.7) | 116(75.3) |  |  |
| Military related | 0 | 7(100.0) |  |  |
| Student | 176(62.0) | 108(38.0) |  |  |
| Unemployed | 26(33.3) | 52(66.7) |  |  |
| Retired | 9(28.1) | 23(71.9) |  |  |
| Full-time Mom/Dad | 26(26.5) | 72(73.5) |  |  |
| Other | 76(35.2) | 140(64.8) |  |  |
| Monthly per capita family income(yuan) | | | 0.000 | 0.098 |
| <3000 | 74(45.4) | 89(54.6) |  |  |
| 3000-6000 | 229(38.5) | 366(61.5) |  |  |
| 6001-9000 | 302(45.0) | 369(55.0) |  |  |
| >9000 | 810(51.1) | 776(48.9) |  |  |
| BMI(kg/m^2^) | | | 0.753 | 0.02 |
| <18.5 | 155(46.3) | 180(53.7) |  |  |
| 18.5-23.9 | 855(47.3) | 954(52.7) |  |  |
| 24-27.9 | 302(47.9) | 328(52.1) |  |  |
| ≥28 | 97(43.9) | 124(56.1) |  |  |

**The correlation coefficient defaults to coefficient of association*

eTable 4 Do you think obesity is a disease for children and adolescents?

|  | Do you think obesity is a disease for children and adolescents | | | P value | Coefficient of association |
| --- | --- | --- | --- | --- | --- |
| Variables | Yes | No | Don't know |  |  |
| Gender | | | | 0.000 | 0.127 |
| Male | 995(76.3) | 168(12.9) | 141(10.8) |  |  |
| Female | 1469(85.9) | 111(6.5) | 131(7.7) |  |  |
| Education | | | | 0.000 | 0.155 |
| Junior high school and below | 62(67.4) | 10(10.9) | 20(21.7) |  |  |
| High school or junior college | 190(68.6) | 32(11.6) | 55(19.9) |  |  |
| Undergraduate or college | 1651(83.0) | 179(9.0) | 158(7.9) |  |  |
| Master's degree and above | 561(85.3) | 58(8.8) | 39(5.9) |  |  |
| Age(years) | | | | 0.006 | 0.084 |
| <18 | 33(61.1) | 10(18,5) | 11(20.4) |  |  |
| 18-29 | 1151(81.6) | 134(9.5) | 125(8.9) |  |  |
| 30-39 | 854(82.4) | 92(8.9) | 91(8.8) |  |  |
| 40-49 | 319(83.1) | 27(7.0) | 38(9.9) |  |  |
| ≥50 | 107(82.3) | 16(12.3) | 7(5.4) |  |  |
| Careers | | | | 0.000 | 0.156 |
| Finance | 254(81.9) | 39(12.6) | 17(5.5) |  |  |
| Education | 348(84.1) | 39(9.4) | 27(6.5) |  |  |
| Health Care | 300(90.4) | 18(5.4) | 14(4.2) |  |  |
| Media,Arts | 115(85.8) | 8(6.0) | 11(8.2) |  |  |
| Industrial Technology Related | 194(81.5) | 24(10.1) | 20(8.4) |  |  |
| Science,information technology related | 206(77.4) | 27(10.2) | 33(12.4) |  |  |
| Agriculture,forestry,fishery and water production | 57(81.4) | 6(8.6) | 7(10.0) |  |  |
| Service industry | 277(80.5) | 24(7.0) | 43(12.5) |  |  |
| Transportation equipment related | 30(78.9) | 3(7.9) | 5(13.2) |  |  |
| Self-employed | 118(76.6) | 14(9.1) | 22(14.3) |  |  |
| Military related | 4(57.1) | 3(7.9) | 0 |  |  |
| Student | 225(79.2) | 34(12.0) | 25(8.8) |  |  |
| Unemployed | 59(75.6) | 4(5.1) | 15(19.2) |  |  |
| Retired | 23(71.9) | 6(18.8) | 3(9.4) |  |  |
| Full-time Mom/Dad | 82(83.7) | 9(9.2) | 7(7.1) |  |  |
| Other | 172(79.6) | 21(9.7) | 23(10.6) |  |  |
| Monthly per capita family income(yuan) | | | | 0.119 | 0.058 |
| <3000 | 123(75.5) | 22(13.5) | 18(11.0) |  |  |
| 3000-6000 | 482(81.0) | 52(8.7) | 61(10.3) |  |  |
| 6001-9000 | 542(80.8) | 60(8.9) | 69(10.3) |  |  |
| >9000 | 1317(83.0) | 145(9.1) | 124(7.8) |  |  |
| BMI(kg/m^2^) | | | | 0.020 | 0.07 |
| <18.5 | 272(78.8) | 35(10.1) | 38(11.0) |  |  |
| 18.5-23.9 | 1503(83.1) | 141(7.8) | 165(9.1) |  |  |
| 24-27.9 | 492(78.1) | 79(12.5) | 59(9.4) |  |  |
| ≥28 | 180(81.4) | 22(10.0) | 19(8.6) |  |  |

**The correlation coefficient defaults to coefficient of association*

eTable 5 What factors do you think may lead to obesity in children and adolescents?

| Variables | Child and adolescent obesity factor score | | | | *P* value | Coefficient of association |
| --- | --- | --- | --- | --- | --- | --- |
|  | 1(%) | 2(%) | 3(%) | 4(%) |  |  |
| Gender | | | | | 0.000 | 0.132 |
| Male | 250(19.2) | 300(23.0) | 263(20.3) | 491(37.7) |  |  |
| Female | 175(10.2) | 281(16.4) | 384(22.4) | 871(50.9) |  |  |
| Education | | | | | 0.000 | 0.17* |
| Junior high school and below | 43(46.7) | 13(14.1) | 17(18.5) | 19(20.7) |  |  |
| High school or junior college | 66(23.8) | 67(24.2) | 65(23.5) | 79(28.5) |  |  |
| Undergraduate or college | 266(13.4) | 372(18.7) | 442(22.2) | 908(45.7) |  |  |
| Master's degree and above | 50(7.6) | 129(19.6) | 123(21.5) | 356(45.2) |  |  |
| Age(years) | | | | | 0.000 | -0.144* |
| <18 | 7(13.0) | 10(18.5) | 12(22.2) | 25(46.3) |  |  |
| 18-29 | 146(10.4) | 239(17.0) | 302(21.4) | 723(51.3) |  |  |
| 30-39 | 147(14.2) | 231(22.3) | 240(23.1) | 419(40.4) |  |  |
| 40-49 | 80(20.8) | 79(20.6) | 70(18.2) | 155(40.4) |  |  |
| ≥50 | 45(34.6) | 22(16.9) | 23(17.7) | 40(30.8) |  |  |
| Careers | | | | | 0.051 |  |
| Finance | 54(17.4) | 64(20.6) | 72(23.2) | 120(38.7) |  |  |
| Education | 44(10.6) | 90(21.7) | 88(21.3) | 192(46.4) |  |  |
| Health Care | 19(5.7) | 36(10.8) | 44(13.3) | 233(70.2) |  |  |
| Media,Arts | 21(15.7) | 20(14.9) | 33(24.6) | 60(44.8) |  |  |
| Industrial Technology Related | 36(15.1) | 52(21.8) | 51(21.4) | 99(41.6) |  |  |
| Science,information technology related | 32(12.0) | 53(19.9) | 66(24.8) | 115(43.2) |  |  |
| Agriculture,forestry,fishery and water production | 15(21.4) | 11(15.7) | 22(31.4) | 22(31.4) |  |  |
| Service industry | 57(16.6) | 76(22.1) | 81(23.5) | 130(27.8) |  |  |
| Transportation equipment related | 7(18.4) | 11(28.9) | 9(23.7) | 11(28.9) |  |  |
| Self-employed | 47(30.5) | 29(18.8) | 29(18.8) | 49(31.8) |  |  |
| Military related | 2(28.6) | 1(14.3) | 3(42.9) | 1(14.3) |  |  |
| Student | 19(6.7) | 41(14.4) | 58(20.4) | 166(58.5) |  |  |
| Unemployed | 17(21.8) | 16(20.5) | 17(21.8) | 28(35.9) |  |  |
| Retired | 16(50.0) | 5(15.6) | 4(12.5) | 7(21.9) |  |  |
| Full-time Mom/Dad | 12(12.2) | 25(25.5) | 22(22.4) | 39(39.8) |  |  |
| Other | 27(12.5) | 51(23.6) | 48(22.2) | 90(41.7) |  |  |
| Monthly per capita family income(yuan) | | | | | 0.245 | 0.017* |
| <3000 | 28(17.2) | 30(18.4) | 33(20.2) | 72(44.2) |  |  |
| 3000-6000 | 100(16.8) | 101(17.0) | 134(22.5) | 260(43.7) |  |  |
| 6001-9000 | 83(12.4) | 133(19.8) | 152(22.7) | 303(45.2) |  |  |
| >9000 | 214(13.5) | 317(20.0) | 328(20.7) | 727(45.8) |  |  |
| BMI(kg/m^2^) | | | | | 0.000 | -0.125* |
| <18.5 | 13(13.5) | 20(20.8) | 16(16.7) | 47(49.0) |  |  |
| 18.5-23.9 | 26(7.0) | 53(14.3) | 84(22.7) | 207(55.9) |  |  |
| 24-27.9 | 81(11.8) | 114(16.5) | 149(21.6) | 345(50.1) |  |  |
| ≥28 | 305(16.4) | 394(21.2) | 398(21.4) | 763(41.0) |  |  |

**The correlation coefficient defaults to coefficient of association*

**Those marked with"*" are coefficient of rank correlation*

eTable 6 What health problems do you think obesity may cause in children and adolescents?

| Variables | Cognitive scores of health problems associated with obesity in children and adolescents | | | | *P* value | Coefficient of association |
| --- | --- | --- | --- | --- | --- | --- |
|  | 0(%) | 1-5(%) | 6-10(%) | >10(%) |  |  |
| Gender | | | | | 0.000 | 0.140 |
| Male | 25(1.9) | 732(56.1) | 292(22.4) | 255(19.6) |  |  |
| Female | 9(0.5) | 774(45.2) | 563(32.9) | 365(21.3) |  |  |
| Education | | | | | 0.000 | 0.079* |
| Junior high school and below | 7(7.6) | 60(65.2) | 15(16.3) | 10(10.9) |  |  |
| High school or junior college | 9(3.2) | 158(57.0) | 69(24.9) | 41(14.8) |  |  |
| Undergraduate or college | 14(0.7) | 974(49.0) | 575(28.9) | 425(21.4) |  |  |
| Master's degree and above | 4(0.6) | 314(47.7) | 196(29.8) | 144(21.9) |  |  |
| Age(years) | | | | | 0.006 | -0.04* |
| <18 | 0(0.0) | 33(61.1) | 9(16.7) | 12(22.2) |  |  |
| 18-29 | 11(0.8) | 692(49.1) | 394(27.9) | 313(22.2) |  |  |
| 30-39 | 12(1.2) | 502(48.4) | 325(31.3) | 198(19.1) |  |  |
| 40-49 | 6(1.6) | 203(52.9) | 95(24.7) | 80(13.1) |  |  |
| ≥50 | 5(1.1) | 76(50.0) | 32(28.4) | 17(20.6) |  |  |
| Careers | | | | | 0.000 | 0.256 |
| Finance | 3(1.0) | 167(53.9) | 79(25.5) | 61(19.7) |  |  |
| Education | 7(1.7) | 215(51.9) | 111(26.8) | 81(19.6) |  |  |
| Health Care | 0(0.0) | 77(23.2) | 116(34.9) | 139(41.9) |  |  |
| Media,Arts | 1(0.7) | 61(45.5) | 50(37.3) | 22(16.4) |  |  |
| Industrial Technology Related | 1(0.4) | 134(56.3) | 69(29.0) | 34(14.3) |  |  |
| Science,information technology related | 4(1.5) | 149(56.0) | 73(27.5) | 40(15.0) |  |  |
| Agriculture,forestry,fishery and water production | 3(4.3) | 35(50.0) | 25(35.7) | 7(10.0) |  |  |
| Service industry | 3(0.9) | 168(48.8) | 100(29.1) | 73(21.2) |  |  |
| Transportation equipment related | 3(7.9) | 18(47.4) | 13(34.2) | 4(10.5) |  |  |
| Self-employed | 2(1.3) | 77(50.0) | 43(27.9) | 32(20.8) |  |  |
| Military related | 0(0.0) | 5(71.4) | 1(14.3) | 1(14.3) |  |  |
| Student | 2(0.7) | 143(50.4) | 75(26.4) | 64(22.5) |  |  |
| Unemployed | 3(3.8) | 48(61.5) | 13(16.7) | 14(17.9) |  |  |
| Retired | 0(0.0) | 24(75.0) | 6(18.8) | 2(6.2) |  |  |
| Full-time Mom/Dad | 1(1.0) | 49(50.0) | 36(36.7) | 12(12.3) |  |  |
| Other | 3(1.4) | 119(55.1) | 53(24.5) | 41(19.0) |  |  |
| Monthly per capita family income(yuan) | | | | | 0.112 |  |
| <3000 | 5(3.1) | 85(52.1) | 47(28.8) | 26(16.0) |  |  |
| 3000-6000 | 5(0.8) | 305(51.3) | 163(27.4) | 122(20.5) |  |  |
| 6001-9000 | 10(1.5) | 327(48.70 | 204(30.4) | 130(19.4) |  |  |
| >9000 | 14(0.9) | 789(49.7) | 441(27.8) | 342(21.6) |  |  |
| BMI(kg/m^2^) | | | | | 0.377 |  |
| <18.5 | 2(2.1) | 55(57.3) | 15(15.6) | 24(25.0) |  |  |
| 18.5-23.9 | 3(0.8) | 192(51.9) | 94(25.4) | 81(21.9) |  |  |
| 24-27.9 | 6(0.9) | 322(46.7) | 199(28.9) | 162(23.5) |  |  |
| ≥28 | 23(1.2) | 937(50.4) | 547(29.4) | 353(19.0) |  |  |

**The correlation coefficient defaults to coefficient of association*

**Those marked with"*" are coefficient of rank correlation*

eTable 7 Do you think obesity is more harmful to children and adolescents than adults?

| Variables | Which is more harmful | | | *P* value | Coefficient of association |
| --- | --- | --- | --- | --- | --- |
|  | children | adults | Same |  |  |
| Gender | | | | 0.000 | 0.115 |
| Male | 532(40.8) | 146(11.2) | 626(48.0) |  |  |
| Female | 579(33.8) | 121(7.1) | 1011(59.1) |  |  |
| Education | | | | 0.000 | 0.117 |
| Junior high school and below | 27(29.3) | 9(9.8) | 56(60.9) |  |  |
| High school or junior college | 77(27.8) | 33(11.9) | 167(60.3) |  |  |
| Undergraduate or college | 701(35.3) | 174(8.8) | 1113(56.0) |  |  |
| Master's degree and above | 306(46.5) | 51(7.8) | 301(45.7) |  |  |
| Age(years) | | | | 0.00 | 0.123 |
| <18 | 16(29.6) | 5(9.3) | 33(61.1) |  |  |
| 18-29 | 439(31.1) | 146(10.3) | 826(58.5) |  |  |
| 30-39 | 424(40.9) | 78(7.5) | 535(51.6) |  |  |
| 40-49 | 175(45.6) | 27(7.0) | 182(47.4) |  |  |
| ≥50 | 58(44.6) | 11(8.5) | 61(46.9) |  |  |
| Careers | | | | 0.000 | 0.305 |
| Finance | 129(41.6) | 25(8.1) | 156(50.3) |  |  |
| Education | 153(37.0) | 39(9.4) | 222(53.6) |  |  |
| Health Care | 160(48.2) | 20(6.0) | 152(45.8) |  |  |
| Media,Arts | 38(28.4) | 13(9.7) | 83(61.9) |  |  |
| Industrial Technology Related | 105(44.1) | 29(12.2) | 104(43.7) |  |  |
| Science,information technology related | 98(36.8) | 27(10.2) | 141(53.0) |  |  |
| Agriculture,forestry,fishery and water production | 29(41.4) | 6(8.6) | 35(50.0) |  |  |
| Service industry | 111(32.3) | 35(10.2) | 198(57.6) |  |  |
| Transportation equipment related | 12(31.6) | 5(13.2) | 21(55.3) |  |  |
| Self-employed | 51(33.1) | 16(10.4) | 87(56.5) |  |  |
| Military related | 3(42.9) | 2(28.6) | 2(28.6) |  |  |
| Student | 95(33.5) | 21(7.4) | 168(59.2) |  |  |
| Unemployed | 25(31.6) | 49(62.0) | 5(6.3) |  |  |
| Retired | 10(31.3) | 1(3.1) | 21(65.6) |  |  |
| Full-time Mom/Dad | 26(26.5) | 4(4.1) | 68(69.4) |  |  |
| Other | 67(31.0) | 19(8.8) | 130(60.2) |  |  |
| Monthly per capita family income(yuan) | | | | 0.004 | 0.08 |
| <3000 | 53(32.5) | 16(9.8) | 94(57.7) |  |  |
| 3000-6000 | 186(31.3) | 61(10.3) | 348(58.5) |  |  |
| 6001-9000 | 233(34.7) | 63(9.4) | 375(55.9) |  |  |
| >9000 | 639(40.3) | 127(8.0) | 820(51.7) |  |  |
| BMI(kg/m^2^) | | | | 0.208 | 0.053 |
| <18.5 | 114(34.0) | 26(7.8) | 195(58.2) |  |  |
| 18.5-23.9 | 668(36.9) | 162(9.0) | 979(54.1) |  |  |
| 24-27.9 | 251(39.8) | 59(9.4) | 320(50.8) |  |  |
| ≥28 | 72(32.6) | 16(7.2) | 133(60.2) |  |  |

**The correlation coefficient defaults to coefficient of association*

eTable 8 What do you think the role of MBS includes?

| Variables | Cognitive scores of the effects of MBS on children and adolescents | | | | *P* value | Coefficient of association |
| --- | --- | --- | --- | --- | --- | --- |
|  | 0(%) | 1-5(%) | 6-10(%) | >10(%) |  |  |
| Gender | | | | | 0.000 | 0.175 |
| Male | 123(9.4) | 862(66.1) | 134(10.3) | 185(14.2) |  |  |
| Female | 65(3.8) | 984(57.5) | 319(18.6) | 343(20.0) |  |  |
| Education | | | | | 0.004 | 0.027* |
| Junior high school and below | 9(9.8) | 60(65.2) | 11(12.0) | 12(13.0) |  |  |
| High school or junior college | 16(5.8) | 185(66.8) | 40(14.4) | 36(13.0) |  |  |
| Undergraduate or college | 115(5.8) | 1209(60.8) | 311(15.6) | 353(17.8) |  |  |
| Master's degree and above | 48(7.3) | 392(59.6) | 91(13.8) | 127(19.3) |  |  |
| Age(years) | | | | | 0.075 | -0.36* |
| <18 | 4(7.4) | 33(61.1) | 7(13.0) | 10(18.5) |  |  |
| 18-29 | 62(4.4) | 876(62.1) | 208(14.8) | 264(18.7) |  |  |
| 30-39 | 81(7.8) | 632(60.9) | 156(15.0) | 168(16.2) |  |  |
| 40-49 | 32(8.3) | 228(59.4) | 55(14.3) | 69(18.0) |  |  |
| ≥50 | 9(6.9) | 77(59.2) | 27(20.8) | 17(13.1) |  |  |
| Careers | | | | | 0.000 | 0.306* |
| Finance | 25(8.1) | 200(64.5) | 42(13.5) | 43(13.9) |  |  |
| Education | 29(8.7) | 263(78.7) | 61(6.3) | 61(6.3) |  |  |
| Health Care | 10(3.0) | 118(35.5) | 69(20.8) | 135(40.7) |  |  |
| Media,Arts | 8(6.0) | 75(56.0) | 31(23.1) | 20(14.9) |  |  |
| Industrial Technology Related | 17(7.1) | 167(70.2) | 30(12.6) | 24(10.1) |  |  |
| Science,information technology related | 25(9.4) | 170(63.9) | 40(15.0) | 31(11.7) |  |  |
| Agriculture,forestry,fishery and water production | 9(12.9) | 42(60.0) | 10(14.3) | 9(12.9) |  |  |
| Service industry | 20(5.8) | 221(64.2) | 37(10.8) | 66(19.2) |  |  |
| Transportation equipment related | 3(7.9) | 30(78.9) | 3(7.9) | 2(5.3) |  |  |
| Self-employed | 4(2.6) | 106(68.8) | 21(13.6) | 23(14.9) |  |  |
| Military related | 1(14.3) | 3(42.9) | 1(14.3) | 2(28.6) |  |  |
| Student | 11(3.9) | 165(58.1) | 43(15.1) | 65(22.9) |  |  |
| Unemployed | 7(9.0) | 56(71.8) | 5(6.4) | 10(12.8) |  |  |
| Retired | 3(9.4) | 21(65.6) | 6(18.8) | 2(6.3) |  |  |
| Full-time Mom/Dad | 6(6.1) | 63(64.3) | 19(19.4) | 10(10.2) |  |  |
| Other | 10(4.6) | 146(67.6) | 35(16.2) | 25(11.6) |  |  |
| Monthly per capita family income(yuan) | | | | | 0.206 |  |
| <3000 | 11(6.7) | 109(66.9) | 20(12.3) | 23(14.1) |  |  |
| 3000-6000 | 42(7.1) | 363(61.0) | 86(14.5) | 104(17.5) |  |  |
| 6001-9000 | 27(4.0) | 426(63.5) | 104(15.5) | 114(17.0) |  |  |
| >9000 | 108(6.8) | 948(59.8) | 243(15.3) | 287(18.1) |  |  |
| BMI(kg/m^2^) | | | | | 0.083 |  |
| <18.5 | 8(8.3) | 57(59.4) | 9(9.4) | 22(22.9) |  |  |
| 18.5-23.9 | 18(4.9) | 232(62.7) | 49(13.2) | 71(19.2) |  |  |
| 24-27.9 | 31(4.5) | 418(60.7) | 111(16.1) | 129(18.7) |  |  |
| ≥28 | 131(7.0) | 1139(61.2) | 284(15.3) | 306(16.5) |  |  |

**The correlation coefficient defaults to coefficient of association*

**Those marked with"*" are coefficient of rank correlation*

eTable 9 If your child is overweight or obese and traditional diet and exercise therapy is not effective, whether you will allow your child to undergo MBS?

| Variables | Yes(%) | No(%) | *P* value | *Coefficient of association* |
| --- | --- | --- | --- | --- |
| Gender | | | 0.000 | 0.09 |
| Male | 682(52.3) | 622(47.7) |  |  |
| Female | 1050(61.4) | 661(38.6) |  |  |
| Education | | | 0.517 |  |
| Junior high school and below | 57(62.0) | 35(38.0) |  |  |
| High school or junior college | 163(58.8) | 114(41.2) |  |  |
| Undergraduate or college | 1148(57.7) | 840(42.3) |  |  |
| Master's degree and above | 364(55.3) | 294(44.7) |  |  |
| Age(years) | | | 0.000 | 0.132 |
| <18 | 38(70.4) | 16(29.6) |  |  |
| 18-29 | 901(63.9) | 509(36.1) |  |  |
| 30-39 | 529(51.0) | 508(49.0) |  |  |
| 40-49 | 198(51.6) | 186(48.4) |  |  |
| ≥50 | 66(50.8) | 64(49.2) |  |  |
| Careers | | | 0.000 | 0.140 |
| Finance | 169(54.5) | 141(45.5) |  |  |
| Education | 221(53.4) | 193(46.6) |  |  |
| Health Care | 221(66.6) | 111(33.4) |  |  |
| Media,Arts | 87(64.9) | 47(35.1) |  |  |
| Industrial Technology Related | 128(53.8) | 110(46.2) |  |  |
| Science,information technology related | 135(50.8) | 131(49.2) |  |  |
| Agriculture,forestry,fishery and water production | 42(60.0) | 28(40.0) |  |  |
| Service industry | 191(55.5) | 153(44.5) |  |  |
| Transportation equipment related | 22(57.9) | 16(42.1) |  |  |
| Self-employed | 95(61.7) | 59(38.3) |  |  |
| Military related | 4(57.1) | 3(42.9) |  |  |
| Student | 203(71.5) | 81(28.5) |  |  |
| Unemployed | 41(52.6) | 37(47.4) |  |  |
| Retired | 13(40.6) | 19(59.4) |  |  |
| Full-time Mom/Dad | 54(55.1) | 44(44.9) |  |  |
| Other | 106(49.1) | 110(50.9) |  |  |
| Monthly per capita family income(yuan) | | | 0.037 | 0.053 |
| <3000 | 102(62.6) | 61(37.4) |  |  |
| 3000-6000 | 359(60.3) | 236(39.7) |  |  |
| 6001-9000 | 398(59.3) | 273(40.7) |  |  |
| >9000 | 873(55.0) | 713(45.0) |  |  |
| BMI(kg/m^2^) | | | 0.000 | 0.485 |
| <18.5 | 71(38.2) | 115(61.8) |  |  |
| 18.5-23.9 | 259(25.5) | 757(74.5) |  |  |
| 24-27.9 | 427(58.3) | 305(41.7) |  |  |
| ≥28 | 975(90.2) | 106(9.8) |  |  |

**The correlation coefficient defaults to coefficient of association*

eTable 10 If you decide to have your child undergo MBS, what age do you think is appropriate?

|  | frequency | Have children | Childless |
| --- | --- | --- | --- |
| Age is not an issue for me | 1208(40) | 385(31.8) | 824(68.2) |
| I will not allow my children to undergo MBS | 1045(34.7) | 500(47.8) | 545(52.2) |
| 6 to 11 years old | 38(1.3) | 16(42.1) | 22(57.9) |
| 12 to 18 years old | 561(18.6) | 235(41.9) | 326(58.1) |
| 19 and 29 years old | 138(4.6) | 63(45.7) | 75(54.3) |
| 30 years old or more | 19(0.6) | 10(52.6) | 9(47.4) |
| Have no idea | 6(0.2) | 2(33.3) | 4(66.7) |
| *P* value | | 0.000  Coefficient of association:0.145 | |

eTable 11 If your child has obesity-related problems between the ages of 7 and 18, and they have a strong desire to have surgery, would you agree to have surgery?

| Variables | Yes(%) | No(%) | *P* value | *Coefficient of association* |
| --- | --- | --- | --- | --- |
| Gender | | | 0.083 | 0.032 |
| Male | 880(67.5) | 424(32.5) |  |  |
| Female | 1205(70.4) | 506(29.6) |  |  |
| Do you have any children | | | 0.000 | 0.097 |
| Yes | 771(63.7) | 440(36.3) |  |  |
| No | 1314(72.8) | 490(27.2) |  |  |
| Education | | | 0.360 | 0.033 |
| Junior high school and below | 63(68.5) | 29(31.5) |  |  |
| High school or junior college | 186(67.1) | 91(32.9) |  |  |
| Undergraduate or college | 1396(70.2) | 592(29.8) |  |  |
| Master's degree and above | 440(66.9) | 218(33.1) |  |  |
| Age(years) | | | 0.000 | 0.129 |
| <18 | 43(79.6) | 11(20.4) |  |  |
| 18-29 | 1058(75.0) | 352(25.0) |  |  |
| 30-39 | 659(63.5) | 378(36.5) |  |  |
| 40-49 | 248(64.6) | 136(35.4) |  |  |
| ≥50 | 77(59.2) | 53(40.8) |  |  |
| Careers | | | 0.000 | 0.122 |
| Finance | 219(70.6) | 91(29.4) |  |  |
| Education | 265(64.0) | 149(36.0) |  |  |
| Health Care | 254(76.5) | 78(23.5) |  |  |
| Media,Arts | 94(70.1) | 40(29.9) |  |  |
| Industrial Technology Related | 161(67.6) | 77(32.4) |  |  |
| Science,information technology related | 179(67.3) | 87(32.7) |  |  |
| Agriculture,forestry,fishery and water production | 54(77.1) | 16(22.9) |  |  |
| Service industry | 228(66.3) | 116(33.7) |  |  |
| Transportation equipment related | 26(68.4) | 12(31.6) |  |  |
| Self-employed | 111(72.1) | 43(27.9) |  |  |
| Military related | 4(57.1) | 3(42.9) |  |  |
| Student | 226(79.6) | 58(20.4) |  |  |
| Unemployed | 52(66.7) | 26(33.3) |  |  |
| Retired | 18(56.3) | 14(43.8) |  |  |
| Full-time Mom/Dad | 56(57.1) | 42(42.9) |  |  |
| Other | 138(63.9) | 78(36.1) |  |  |
| Monthly per capita family income(yuan) | | | 0.230 | 0.038 |
| <3000 | 118(72.4) | 45(27.6) |  |  |
| 3000-6000 | 425(71.4) | 170(28.6) |  |  |
| 6001-9000 | 470(70.0) | 201(30.0) |  |  |
| >9000 | 1072(67.6) | 514(32.4) |  |  |
| BMI(kg/m^2^) | | | 0.000 | 0.466 |
| <18.5 | 81(47.4) | 90(52.6) |  |  |
| 18.5-23.9 | 294(35.5) | 535(64.5) |  |  |
| 24-27.9 | 502(69.1) | 225(30.9) |  |  |
| ≥28 | 1208(93.5) | 80(6.5) |  |  |

**The correlation coefficient defaults to coefficient of association*

**Those marked with"*" are coefficient of rank correlation*

eFigures in Supplementary Files

efigure 1

efigure 2

efigure 3

efigure 4

Appendix 1: Questionnaire

Perceptions and attitudes of Chinese residents towards obesity and metabolic and bariatric surgery for weight loss in children and adolescents: a national survey

1. Your age: ____years old

2. Your gender

(1) Male
(2) Female

3. Your education

(1) Junior high school and below
(2) High school or junior college
(3) Bachelor's degree or college
(4) Master and above

4. Your Marital Status

(1) Unmarried
(2) Married
(3) Divorce
(4) Bereaved spouse

5. Your occupation

(1)	Finance

(2)	Education

(3)	Healthcare

(4)	Media, Art

(5)	Industrial Technology Related

(6)	Science, information technology-related

(7)	Agriculture, forestry, animal husbandry, fishery, water production

(8)	Service industry

(9)	Transportation equipment related

(10)	Self-employed

(11)	Military Related

(12)	Students

(13)	Unemployed

(14)	Retirement

(15)	Full-time Mom/Dad

(16)	Other

6. Monthly per capita family income (Chinese Yuan)

(1) <3000
(2) 3001~6000
(3) 6001~9000
(4) ＞9001

7. Your Height	(cm); Weight	(kg)

8. Evaluation of your own body type.

(1) Thin
(2) Standard
(3) Overweight
(4) Obesity

9. Whether you have received weight reduction metabolic and bariatric surgery(MBS)?

(1) Yes
(2) No

10. Do you have children?

(1) No children
(2) One child
(3) Have two or more children

*For questions 11-17, if you have more than one child, please fill in the information of a child aged 7-18 years old as a priority; if not in this range, then choose any one child to fill in.*

11. The gender of your children

(1) Male
(2) Female

12. Your child's age

13. Your children's education

(1) Elementary school or below
(2) Junior High School
(3) High School
(4) Bachelor's degree or above

14. Your child's permanent place of residence.

(1) Overseas: Foreign countries / Hong Kong, Macao and Taiwan region
(2) Within Province

15. Your child's Height	(cm) weight	(kg)

Your child's weight at birth	(kg)(2.5-4.0 normal)

16. Your child's current life pattern at this stage.

(1) Living with parents
(2) Boarding at school
(3) Living with grandparents or other relatives
(4) Living with a host family
(5) Living alone or with a partner or friend

17. Evaluation of children's body type.

(1) Thin
(2) Standard
(3) Overweight
(4) Obesity

Perceptions and attitudes towards obesity and weight loss in children and adolescents

18. Do you know about body mass index(BMI)?

(1) Understanding
(2) Don't understand

19. Do you know how to calculate body mass index(BMI)?

(1) Yes
(2) No

20. Do you think obesity is a disease for children and adolescents?

(1) Yes, I think so
(2) No, I don't think so
(3) Not sure

21. What factors do you think may lead to obesity in children and adolescents? (Multiple choice)

(1) Genetic factors
(2) Diet and lifestyle
(3) Sleep and psychosomatic factors
(4) Illness and medication use
(5) Other

22. Which health problems do you think obesity may lead to in children and adolescents? (Multiple choice)

(1) Sleep apnea, snoring, drowsiness, easy fatigue, loss of concentration and memory
(2) Delayed intellectual and physical development
(3) Precocious puberty or delayed development of sexual organs
(4) Irregular menstruation and polycystic ovary syndrome
(5) Mental illnesses such as depression and mental anxiety
(6) Cardiovascular and cerebrovascular diseases (hypertension, heart disease)
(7) Gastroesophageal reflux disease(acid reflux, heartburn)
(8) Type 2 diabetes and insulin resistance (hyperglycemia)
(9) Tumor or cancer
(10) Osteoporosis and fractures
(11) Fatty liver
(12) Eczema, acanthosis nigricans and other skin problems
(13) Kidney disease
(14) No major impact on health

23. Do you think obesity is more harmful to children and adolescents than adults?

(1) Childhood and adolescent obesity are more dangerous

(2) Adult obesity is more harmful

(3) Just as harmful

24. What do you think are the most appropriate weight loss interventions for children and adolescents to receive? (Multiple choice)

(1) Dieting

(2) Movement

(3) Adjustment of work and rest

(4) Weight loss pills

(5) metabolic and bariatric surgery(MBS)

(6) Psychological guidance

(7) Liposuction

(8) Meal replacement

(9) Chinese medicine (acupuncture, tui na, cupping, etc.)

(10) Other

(11) Children and adolescents do not need to lose weight

Perceptions and attitudes towards metabolic and bariatric surgery (MBS) for children and adolescents

25. What do you think about the role of metabolic and bariatric surgery for children and adolescents include(multiple choice)

(1) Weight loss
(2) Prevent and improve sleep apnea, snoring, drowsiness, easy fatigue, concentration and	memory loss
(3) Prevention and improvement of mental and physical developmental delays
(4) Prevent and improve precocious puberty or delayed development of sexual organs
(5) Prevention and improvement of irregular menstruation and polycystic ovary syndrome
(6) Prevent and improve mental illnesses such as depression and mental anxiety
(7) Prevent and improve cardiovascular diseases (hypertension, heart disease,)
(8) Prevention and improvement of gastroesophageal reflux disease(acid reflux, heartburn)
(9) Prevent and improve type 2 diabetes and insulin resistance(hyperglycemia)
(10) Prevention of tumors or cancer
(11) Prevent and improve osteoporosis and fractures
(12) Prevent and improve fatty liver
(13) Prevent and improve kidney disease
(14) Prevent and improve skin problems such as eczema and acanthosis nigricans
(15) I don't think it works

26. If your child is overweight or obese and traditional diet-exercise treatment is not effective, will you let your child undergo weight reduction metabolic and bariatric surgery?

(1) Yes, undergo surgery
(2) Still no surgery

27. If you decide to have your child undergo metabolic and bariatric surgery, what age do you think would be appropriate?

(1) l think the age at	is more appropriate
(2) Age is not a problem for me
(3) I will not allow my children to undergo metabolic and bariatric surgery

28. If your child has obesity-related problems between the ages of 7 and 18, and they have a strong desire to undergo metabolic and bariatric surgery, would you agree to undergo surgery?

(1) Yes
(2) No

29. What do you think the benefits of undergoing metabolic and bariatric surgery will bring to children and adolescents?(Multiple choice)

(1) Weight loss

(2) Improving disease(e.g., reducing drug use)

(3) Increase self-confidence/improve self-image

(4) Improve academic performance

(5) Perform better in interpersonal relationships

(6) Other

(7) I will not allow my children to undergo metabolic and bariatric surgery

30. If you have concerns about children and adolescents undergoing metabolic and bariatric surgery, what are the possible reasons?(Multiple choice)

(1) Children and adolescents are too young
(2) Insufficient knowledge of the surgery
(3) The procedure is not considered safe and reliable enough
(4) Thinking that the desired weight loss will not be achieved after surgery
(5) Fear of weight regain after surgery
(6) Fear of post-operative complications or other adverse effects (malnutrition, developmental effects)
(7) Cost, economic status and other factors
(8) Other

31. Before doing this questionnaire, have you ever heard of treating obesity and metabolic diseases through surgery?

(1) Never heard of it
(2) Yes, I have heard

32. If you have heard of it, from which of the following sources did you hear about it?(Multiple choice)

(1) TV
(2) Magazine
(3) Website
(4) WeChat
(5) Short video
(6) Microblog
(7) Family, friends or classmates
(8) Health care workers
(9) School education
(10) Government propaganda
(11) Academic literature
(12) Others
(13) Don't remember

33. What are the ways you would like to know the information related to weight reduction and metabolic surgery?(Multiple choice)

(1) Television
(2) Magazines
(3) Websites
(4) WeChat
(5) Short videos
(6) Microblogs
(7) Family, friends or classmates
(8) Health care professionals
(9) School education
(10) Government propaganda
(11) Academic literature
(12) Others
(13) Don't want to know

Appendix 2: Checklist for Reporting Results of Internet E-Surveys (CHERRIES)

| Item Category | Checklist Item | Explanation | Page Number |
| --- | --- | --- | --- |
| Design | | | |
|  | Describe survey design | Describe target population, sample frame. Is the sample a convenience sample? (In “open” surveys this is most likely.) | 5 |
| IRB (Institutional Review Board) approval and informed consent process | | | |
|  | IRB approval | Mention whether the study has been approved by an IRB. | 5 |
|  | Informed consent | Describe the informed consent process. Where were the participants told the length of time of the survey, which data were stored and where and for how long, who the investigator was, and the purpose of the study? | 5 |
|  | Data protection | If any personal information was collected or stored, describe what mechanisms were used to protect unauthorized access. | 5 |
| Development and pre-testing | | | |
|  | Development and testing | State how the survey was developed, including whether the usability and technical functionality of the electronic questionnaire had been tested before fielding the questionnaire. | 5 |
| Recruitment process and description of the sample having access to the questionnaire | | | |
|  | Open survey versus closed survey | An “open survey” is a survey open for each visitor of a site, while a closed survey is only open to a sample which the investigator knows (password-protected survey). | 5 |
|  | Contact mode | Indicate whether or not the initial contact with the potential participants was made on the Internet. (Investigators may also send out questionnaires by mail and allow for Web-based data entry.) | 5 |
|  | Advertising the survey | How/where was the survey announced or advertised? Some examples are offline media (newspapers), or online (mailing lists – If yes, which ones?) or banner ads (Where were these banner ads posted and what did they look like?). It is important to know the wording of the announcement as it will heavily influence who chooses to participate. Ideally the survey announcement should be published as an appendix. | 5 |
| Survey administration | | | |
|  | Web/E-mail | State the type of e-survey (eg, one posted on a Web site, or one sent out through e-mail). If it is an e-mail survey, were the responses entered manually into a database, or was there an automatic method for capturing responses? | 6 |
|  | Context | Describe the Web site (for mailing list/newsgroup) in which the survey was posted. What is the Web site about, who is visiting it, what are visitors normally looking for? Discuss to what degree the content of the Web site could pre-select the sample or influence the results. For example, a survey about vaccination on a anti-immunization Web site will have different results from a Web survey conducted on a government Web site | 6 |
|  | Mandatory/voluntary | Was it a mandatory survey to be filled in by every visitor who wanted to enter the Web site, or was it a voluntary survey? | 5 |
|  | Incentives | Were any incentives offered (eg, monetary, prizes, or non-monetary incentives such as an offer to provide the survey results)? | 5 |
|  | Time/Date | In what timeframe were the data collected? | 5 |
|  | Randomization of items or questionnaires | To prevent biases items can be randomized or alternated. |  |
|  | Adaptive questioning | Use adaptive questioning (certain items, or only conditionally displayed based on responses to other items) to reduce number and complexity of the questions. | 5 |
|  | Number of Items | What was the number of questionnaire items per page? The number of items is an important factor for the completion rate. | 5 |
|  | Number of screens (pages) | Over how many pages was the questionnaire distributed? The number of items is an important factor for the completion rate. | 5 |
|  | Completeness check | It is technically possible to do consistency or completeness checks before the questionnaire is submitted. Was this done, and if “yes”, how (usually JAVAScript)? An alternative is to check for completeness after the questionnaire has been submitted (and highlight mandatory items). If this has been done, it should be reported. All items should provide a non-response option such as “not applicable” or “rather not say”, and selection of one response option should be enforced. | 5-6 |
|  | Review step | State whether respondents were able to review and change their answers (eg, through a Back button or a Review step which displays a summary of the responses and asks the respondents if they are correct). | 5-6 |
| Response rates | | | |
|  | Unique site visitor | If you provide view rates or participation rates, you need to define how you determined a unique visitor. There are different techniques available, based on IP addresses or cookies or both. |  |
|  | View rate (Ratio of unique survey visitors/unique site visitors) | Requires counting unique visitors to the first page of the survey, divided by the number of unique site visitors (not page views!). It is not unusual to have view rates of less than 0.1 % if the survey is voluntary. |  |
|  | Participation rate (Ratio of unique visitors who agreed to participate/unique first survey page visitors) | Count the unique number of people who filled in the first survey page (or agreed to participate, for example by checking a checkbox), divided by visitors who visit the first page of the survey (or the informed consents page, if present). This can also be called “recruitment” rate. |  |
|  | Completion rate (Ratio of users who finished the survey/users who agreed to participate) | The number of people submitting the last questionnaire page, divided by the number of people who agreed to participate (or submitted the first survey page). This is only relevant if there is a separate “informed consent” page or if the survey goes over several pages. This is a measure for attrition. Note that “completion” can involve leaving questionnaire items blank. This is not a measure for how completely questionnaires were filled in. (If you need a measure for this, use the word “completeness rate”.) | 6 |
| Preventing multiple entries from the same individual | | | |
|  | Cookies used | Indicate whether cookies were used to assign a unique user identifier to each client computer. If so, mention the page on which the cookie was set and read, and how long the cookie was valid. Were duplicate entries avoided by preventing users access to the survey twice; or were duplicate database entries having the same user ID eliminated before analysis? In the latter case, which entries were kept for analysis (eg, the first entry or the most recent)? |  |
|  | IP check | Indicate whether the IP address of the client computer was used to identify potential duplicate entries from the same user. If so, mention the period of time for which no two entries from the same IP address were allowed (eg, 24 hours). Were duplicate entries avoided by preventing users with the same IP address access to the survey twice; or were duplicate database entries having the same IP address within a given period of time eliminated before analysis? If the latter, which entries were kept for analysis (eg, the first entry or the most recent)? | 6 |
|  | Log file analysis | Indicate whether other techniques to analyze the log file for identification of multiple entries were used. If so, please describe. |  |
|  | Registration | In “closed” (non-open) surveys, users need to login first and it is easier to prevent duplicate entries from the same user. Describe how this was done. For example, was the survey never displayed a second time once the user had filled it in, or was the username stored together with the survey results and later eliminated? If the latter, which entries were kept for analysis (eg, the first entry or the most recent)? |  |
| Analysis | | | |
|  | Handling of incomplete questionnaires | Were only completed questionnaires analyzed? Were questionnaires which terminated early (where, for example, users did not go through all questionnaire pages) also analyzed? | 6 |
|  | Questionnaires submitted with an atypical timestamp | Some investigators may measure the time people needed to fill in a questionnaire and exclude questionnaires that were submitted too soon. Specify the timeframe that was used as a cut-off point, and describe how this point was determined. | 6 |
|  | Statistical correction | Indicate whether any methods such as weighting of items or propensity scores have been used to adjust for the non-representative sample; if so, please describe the methods. |  |
